# The evolutionary conserved proteins CEP90, FOPNL, and OFD1 recruit centriolar distal appendage proteins to initiate their assembly

**DOI:** 10.1371/journal.pbio.3001782

**Published:** 2022-09-07

**Authors:** Pierrick Le Borgne, Logan Greibill, Marine Hélène Laporte, Michel Lemullois, Khaled Bouhouche, Mebarek Temagoult, Olivier Rosnet, Maeva Le Guennec, Laurent Lignières, Guillaume Chevreux, France Koll, Virginie Hamel, Paul Guichard, Anne-Marie Tassin

**Affiliations:** 1 Université Paris-Saclay, CEA, CNRS, Institute for Integrative Biology of the Cell (I2BC), Gif-sur-Yvette, France; 2 University of Geneva, Section of Biology, Department of Molecular and Cellular Biology, Geneva, Switzerland; 3 Imagerie-Gif Light facility, Université Paris-Saclay, CEA, CNRS, Institute for Integrative Biology of the Cell (I2BC), Gif-sur-Yvette, France; 4 Centre de Recherche en Cancérologie de Marseille (CRCM), Aix Marseille Univ, CNRS, INSERM, Institut Paoli-Calmettes, Marseille, France; 5 ProteoSeine@IJM, Université de Paris/CNRS, Institut Jacques Monod, Paris, France; Rheinische Friedrich-Wilhelms-Universitat Bonn, GERMANY

## Abstract

In metazoa, cilia assembly is a cellular process that starts with centriole to basal body maturation, migration to the cell surface, and docking to the plasma membrane. Basal body docking involves the interaction of both the distal end of the basal body and the transition fibers/distal appendages, with the plasma membrane. Mutations in numerous genes involved in basal body docking and transition zone assembly are associated with the most severe ciliopathies, highlighting the importance of these events in cilium biogenesis. In this context, the ciliate *Paramecium* has been widely used as a model system to study basal body and cilia assembly. However, despite the evolutionary conservation of cilia assembly events across phyla, whether the same molecular players are functionally conserved, is not fully known. Here, we demonstrated that CEP90, FOPNL, and OFD1 are evolutionary conserved proteins crucial for ciliogenesis. Using ultrastructure expansion microscopy, we unveiled that these proteins localize at the distal end of both centrioles/basal bodies in *Paramecium* and mammalian cells. Moreover, we found that these proteins are recruited early during centriole duplication on the external surface of the procentriole. Functional analysis performed both in *Paramecium* and mammalian cells demonstrate the requirement of these proteins for distal appendage assembly and basal body docking. Finally, we show that mammalian centrioles require another component, Moonraker (MNR), to recruit OFD1, FOPNL, and CEP90, which will then recruit the distal appendage proteins CEP83, CEP89, and CEP164. Altogether, we propose that this OFD1, FOPNL, and CEP90 functional module is required to determine in mammalian cells the future position of distal appendage proteins.

## Abstract 2

CEP90, FOPNL and OFD1 form an evolutionary conserved module which promotes the assembly of centriolar distal appendages. This study uses ultrastructure expansion microscopy to reveal the recruitment of this module on early-born procentrioles to in turn recruit centriolar distal appendage proteins, proposing that this dictates the future location of distal appendages.

## Introduction

The centrosome is the main microtubule-organizing center in most animal cells. It is composed of 2 centrioles, microtubule-based organelles displaying a 9-fold symmetry, surrounded by layers of pericentriolar material [1–3]. A structural asymmetry between the 2 centrioles is observed, the mother one bearing distal and subdistal appendages, while the daughter lacks these appendages [1]. In resting cells, the mother centriole converts into a basal body (BB), which functions as a platform to template the assembly of a cilium. Cilia can be found at the cell surface either unique or in multiple copies, motile or nonmotile [4]. Although well conserved throughout the evolution [5], cilia ultrastructure exhibits variations between cell types in a given organism and between organisms [6–9]. In all cases, cilia possess mechano- and chemo-sensitivity properties. In addition, motile cilia beat to generate either a flow such as cerebral fluid (brain ventricle), mucus (respiratory tract), or the forces required to propulse the cells (spermatozoid, ovum, unicellular organisms) [4].

The cilia assembly process, also called ciliogenesis, is a multistep process involving 4 major events: BB duplication, migration to the cell surface, membrane anchoring of the BB via the distal appendages, and ciliary growth. The conservation of this sequence of events in most phyla is paralleled by an important conservation of the proteins involved [5,10]. The BB anchoring step requires the tethering of the distal appendages, which in mammalian cells contain CEP83, CEP89, SCLT1, FBF1, and CEP164 [11–14], to a membrane. This membrane could be either Golgi apparatus–derived vesicles that fuse together to form the ciliary shaft, as in most metazoan cells, or the plasma membrane directly as in the unicellular organisms *Paramecium* and in some mammalian cell types such as the immunological synapse [15]. The interaction of the BB with the membrane leads to the formation of the transition zone (TZ), bridging the BB to the axoneme, which has recently been recognized to act as a diffusion barrier between the intracellular space and the cilium, defining the ciliary compartment [16,17]. Mutations in genes encoding proteins localized at the BB distal end, distal appendages, or the TZ lead to various syndromes in humans, called ciliopathies (Oro-facial-digital, Nephronophtysis, Joubert, Jeune), reinforcing the importance to understand how these structures and their associated proteins control ciliogenesis [13,18–21].

*Paramecium tetraurelia* is a free-living unicellular organism, easy to cultivate that bear at its surface ca. 4,000 cilia. The corresponding BB are arranged in longitudinal rows, and their polarity is marked by the asymmetrical organization of their associated appendages [22,23]. In *Paramecium*, BB organization is highly precise, with regions composed of units with singlet or doublet BB. Units with doublets BB can display either 2 ciliated BB or a ciliated and an unciliated one [22]. Interestingly, the TZ matures biochemically and structurally between the 2 states [24]. Unlike metazoa, there is no centriolar stage in *Paramecium*: new BB develop from the docked ones. Once duplicated, they tilt up and anchor directly at the surface. Due to the high number of BB at the cell surface, a defect in BB anchoring is easily detected in *Paramecium* by immunofluorescence, making of *Paramecium* an outstanding model to characterize new partners involved in BB anchoring.

The centriolar protein FOPNL/FOR20 together with OFD1 belong to the family of FOP-related proteins, displaying a TOF (33 residues at the N terminus of TON1, OFD1, and FOP) and a LisH (Lis homology domain) domains; the latter one is known to be involved in homodimer formation [25]. Previous work highlighted a role for FOPNL in ciliogenesis, since its depletion in mammalian cells inhibits cilia formation [26] and FOPNL knockout (KO) mice show embryonic lethality at about E11.5 with an important decrease of cilia in the embryonic node. As a consequence, the embryos show left-right symmetry defects [27]. However, the dual localization of FOPNL, at both centrioles and centriolar satellites, prevented determining which pool of FOPNL is involved in ciliogenesis, since centriolar satellites are also involved in cilia formation [26,28]. Interestingly, the depletion of the FOPNL ortholog in *Paramecium* led to the inhibition of BB distal end assembly and, as a consequence, blocks the BB docking process, suggesting that FOPNL could also be involved in centriole distal end formation in mammalian cells [29]. Since *Paramecium* does not have centriolar satellites as determined by the absence of both PCM1 and granular structures (personal observations) hinting for a direct role of FOPNL at the level of BB rather than at centriolar satellite. Further studies in human cells strengthened this hypothesis since FOPNL was shown to interact directly with MNR/OFIP/KIAA0753 in a complex comprising the distal centriolar protein OFD1, the presence of FOPNL increasing the interaction between MNR and OFD1 [21]. In mammals, OFD1 controls centriole length and is required for distal appendage formation [18], while in *Paramecium*, it is necessary for distal end assembly and BB docking similarly to FOPNL.

Here, in order to fully characterize the evolutionary conserved function of FOPNL in BB docking and distal appendages assembly, we undertook the identification of proteins in proximity of FOPNL/FOR20 by BioID in human cells. Among its potential interactants, we focused our study on CEP90/PIBF1, since its encoding gene was found mutated in some ciliopathies [30,31]. Moreover, CEP90 is evolutionarily conserved from protists to human, as OFD1 and FOPNL, despite being absent in some groups as drosophilidae or rhabditidae. Finally, Kumar and colleagues identified the DISCO complex composed of OFD1, MNR, and CEP90 as important for distal appendages formation and proper ciliogenesis [32]. Despite this convergent information, the role of FOPNL at centrioles and its relationship with other members of the centriolar distal complex such as DISCO remained unknown.

Functional analyses were performed both in *Paramecium* and in mammalian cells. Using RNA interference (RNAi), we showed the involvement of CEP90 in BB anchoring as well as unveiled the interdependent recruitment of CEP90 with OFD1 and FOPNL/FOR20 to build the distal end of the BB in *Paramecium*. Furthermore, using ultrastructure expansion microscopy (U-ExM), we uncovered that CEP90, OFD1, and FOPNL localize in *Paramecium* at the distal end of BB in a 9-fold symmetry. Similarly and consistently to the recent work [32], we confirmed in mammalian cells that CEP90 and OFD1 localize subdistally in a 9-fold symmetry and are required, with FOPNL to recruit distal appendage proteins. But, in contrast to Kumar and colleagues [32], MNR, OFD1, and CEP90 are recruited to procentrioles as soon as their formation starts. Next, we took advantage of a microtubule displacement assay in mammalian cells, whereby overexpressed MNR relocates to cytoplasmic microtubules, to analyze protein recruitment and complex formation [21,32]. Consistently, we found that overexpressed MNR recruits overexpressed OFD1, FOPNL, and CEP90 to microtubules, suggesting that they can interact. In addition, MNR and CEP90 coexpression allows the recruitment of endogenous OFD1 as well as the distal appendage proteins CEP83, CEP89, and CEP164 on microtubules. The early recruitment of the evolutionary conserved proteins FOPNL, OFD1, and CEP90 on procentriole together with the recruitment of distal appendage proteins on microtubules of cells coexpressing MNR and CEP90 led us to propose that this complex predetermine the future distal appendage location in mammalian cells 1 or 2 cell cycles before distal appendage assembly.

## Results

To discover novel proteins involved in the BB anchoring process, BioID was performed using FOPNL as a bait in human cells. To do so, Flp-In HEK293 cells expressing myc-BirA*-FOPNL under the control of the tetracycline repressor were generated. Centrosomal biotinylated proteins were purified on streptavidin beads and analyzed by mass spectrometry. Candidates were ranked according to their fold change and *p*-value (S1A and S1B Fig). Forty-eight proteins were identified with a fold change >5 and a *p*-value < 0.05 (S1 Table), and 2 main protein networks were generated using STRING DB to further analyze the data. Consistently, one displayed centriolar satellite proteins as expected from the localization of FOPNL and the other one corresponded to nuclear enriched proteins (S1B and S1C Fig), some of them being involved in chromatin organization as previously observed [33]. Importantly, several known BB anchoring proteins such as Talpid3/KIAA0586 [34] and OFIP/MNR/KIAA0753 [21] were enriched in our mass spectrometry, thus validating our approach (see S1 Table). In addition, some novel potential interactors were found. We focused further our functional analysis on the candidate centriolar protein CEP90/PIBF1 as (i) *CEP90* gene was found mutated in some ciliopathies [30,31]; and (ii) *CEP90* is well conserved from protists to mammals, despite being absent in some phyla.

To validate the putative interaction of FOPNL and CEP90, we performed co-immunoprecipitation experiments on Hela Kyoto TransgeneOmics cells expressing GFP-tagged mouse FOPNL [21]. We found that GFP-FOPNL was able to interact with endogenous OFD1 and CEP90 (S1D Fig).

### Paramecium CEP90-GFP localizes to basal body

To further study the novel interactions found in mammalian cells and to test their evolutionary conservation, we turned to *Paramecium*, a well-established model to decipher the BB anchoring process. We first searched for *CEP90* homologs in the *Paramecium tetraurelia* genome and found 2 *CEP90* homologs, *CEP90a* and *CEP90b*, derived from the last whole genome duplication [35]. RNAseq analysis of the *P*. *tetraurelia* transcriptome during vegetative growth revealed that *CEP90a* is 24× more expressed than *CEP90b* (paramecium.i2bc.paris-saclay.fr) [36]. Therefore, we decided to focus on CEP90a for the rest on this study.

**Fig 1:**
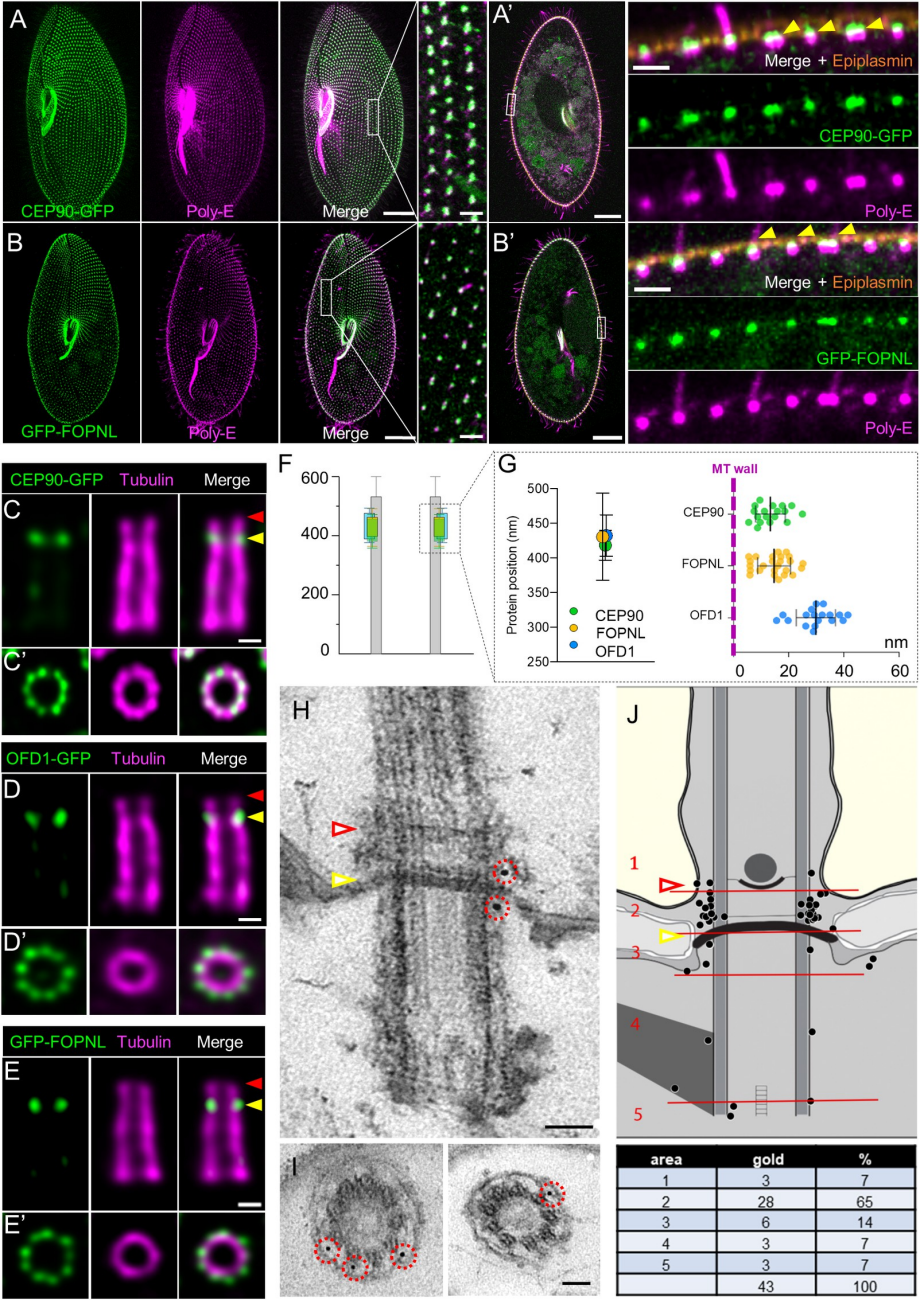
Localization of CEP90 GFP, GFP-FOPNL, and OFD1-GFP in *Paramecium*. **(A-B’**) Labelling of CEP90-GFP (**A, A’**) and GFP-FOPNL (**B, B’**) *Paramecium* transformants using the polyclonal anti-polyglutamylated tubulin antibodies (poly-E, magenta) and anti-epiplasmin (orange). (**A, B**) Projection of confocal sections through the ventral surface. On the ventral side (**A, B**), the green emitted fluorescence of GFP overlapped with the anti-poly-E labeling on all BB, as shown on the magnification on the right panel. Scale bars = 20 μm and 2 μm (magnification). (**A’, B’**) Projection of confocal sections at the intracytoplasmic level. BB at the cell surface showed that the GFP signal (green) is located at the distal part of all BB (magenta) at the level of the epiplasm layer (orange) for both CEP90-GFP and GFP-FOPNL. The yellow arrowheads indicate the level of the epiplasm layer representing the frontier between the BB and the TZ. Scale bars = 20 μm and 1.7 μm (magnification) **(C-G**) Cortex from transformants expressing CEP90-GFP, OFD1-GFP, and GFP-FOPNL were purified and expanded using the U-ExM technique. Gullet cortical fragments displaying ciliated BB were selected since U-ExM gave better results in this region. Anti-GFP antibodies (green) and anti-tubulin antibodies (magenta) were used to label, respectively, the fusion protein and the BB microtubule wall. (**C-E’**) Labeling of the fusion protein on BB observed longitudinally (upper panel) or top view (lower panel). CEP90-GFP (**C**), OFD1-GFP (**D**), and GFP-FOPNL (**E**) BB display the GFP staining close to the microtubule wall of the BB (magenta) and slightly underneath the distal end staining of the anti-tubulin signal. Yellow and red arrowheads indicate the level of the epiplasm layer and the distal part of the TZ, respectively. Top view images demonstrate that the GFP signal is organized in a 9-fold symmetry (**C’, D’, E’**). Scale bar = 100 nm. (**F, G**) The distance to the proximal end of microtubule wall and the protein of interest were quantified and statistically analyzed (***p < 0.0001, one-way ANOVA followed by Tukey’s post hoc test). *N* = 14 (CEP90), 15 (FOPNL), 9 (OFD1) BB from 2 independent experiments. Average +/− SD: CEP90 = 418.4 +/− 21.7 nm; FOPNL = 430.6 +/− 62.6 nm; OFD1 = 432.3 +/− 29.9 nm. Source data can be found in S1 Data. (**H-J**) Representative EM images of the immunolocalization of CEP90-GFP protein on BB revealed by anti-GFP antibodies. (**H, I**) Longitudinal view (**H**) and transverse sections of BBs at the level of the intermediate plate (**I**). Note that gold particles are located close to the TZ or at the level of the intermediate plate. Scale bar = 100 nm. (**J**) BB diagrams recapitulating the localization of gold beads in the 5 different zones indicated (1–5). Quantification of gold particle number counted on 35 BB in 2 independent experiments. Note that most of the beads were localized at the TZ level between the axosomal plate and the terminal plate, known to be the place where the transition fibers emerge from the microtubule doublet. The yellow and red arrowheads indicate the level of the epiplasm layer and the distal part of the TZ, respectively. BB, basal body; EM, electron microscopy; TZ, transition zone; U-ExM, ultrastructure expansion microscopy.

To ascertain the localization of CEP90 in *Paramecium*, we expressed CEP90a-GFP under the control of the constitutive calmodulin gene-regulatory elements. After transformation, rows of GFP dots were observed on transformants displaying a wild-type growth rate and phenotype (Fig 1A). To confirm that this localization pattern corresponds to BB, we costained with tubulin poly-glutamylation antibodies (poly-E), a known BB marker [24]. Consistently, we found that CEP90a-GFP decorates the BB (Fig 1A) and that this staining is reminiscent to GFP-FOPNL (Fig 1B) and OFD1-GFP staining [29,37], as expected for a proximity partner. A careful observation of the GFP staining pattern relative to the BB revealed that all BB are decorated, i.e., ciliated and unciliated (Fig 1A, magnification), unlike the GFP-tagged MKS and NPHP protein complex, which stained only ciliated BB [24]. Double labelling of CEP90a-GFP cells using poly-E antibodies and anti-epiplasm [38], a superficial cytoskeleton layer, revealed that the CEP90a-GFP staining is located at the level of the epiplasm layer, in which the distal ends of the BB are embedded. This result demonstrates that CEP90 localization is restricted to the distal ends of anchored BB (Fig 1A’, magnification, yellow arrowhead) similarly to GFP-FOPNL (Fig 1B’, magnification, yellow arrowhead).

### CEP90, FOPNL, and OFD1 localize at the basal body distal ends in a 9-fold symmetry in *Paramecium*

To characterize more precisely the localization of CEP90 together with FOPNL and OFD1, we turned to U-ExM, a super-resolution method based on the physical expansion of the biological sample in a swellable polymer [39,40]. Since *Paramecium* is 150 μm long and 50 μm thick, U-ExM was performed on isolated cortex sheets [41] from cells expressing GFP-FOPNL, CEP90a-GFP, or OFD1-GFP to allow a nanoscale localization of these proteins within the structure (Fig 1C–1G). During cortex purification, we observed by electron microscopy (EM) ciliary shedding just above the TZ (S2A Fig, white arrowhead), as it is known to occur in physiological conditions [24,42]. Double stained longitudinally oriented BB show that in CEP90a-GFP paramecia, the GFP staining was located either at the top of the BB assessed by the anti-tubulin staining (S2B Fig, yellow arrowhead) or slightly underneath (S2B Fig, white arrowhead). This observed difference could be associated with the ciliation status, since ciliated BB showed a longer TZ with extended microtubule doublets than unciliated ones (S2A Fig) [23,24]. Therefore, we propose that the staining is found at the proximal part of the TZ (Figs 1C–1E and S2B–S2E, green arrowhead) on all BB and the observed tubulin staining above the GFP staining might be associated to ciliated BB (Figs 1C–1E and S2B–S2E, magenta arrowhead) and would correspond to the elongation of the TZ that occurs during ciliogenesis. Longitudinally oriented BB stained for OFD1-GFP (Figs 1D, S2C and S2E) and GFP-FOPNL (Figs 1E and S2D) show a similar pattern than CEP90a-GFP, suggesting that these 3 proteins localized at the end of the BB or at the proximal part of the TZ (Fig 1C–1F). Moreover, analysis of the protein’s positions along the microtubule wall indicated that all 3 proteins localize at the same average position at around 425 nm from the proximal extremity (Fig 1F and 1G). Observations on top-viewed BB showed that the 3 proteins were organized in a 9-fold symmetry, close to the microtubule wall but externally (Fig 1C’, 1D’ and 1E’). Quantification of the distance between both GFP and tubulin maximal intensity signal of the rings revealed that CEP90a-GFP, OFD1-GFP, and GFP-FOPNL localized close to the microtubule wall with CEP90 and FOPNL appearing at around 15 nm away from the microtubule wall, while OFD1 was at around 30 nm, suggesting that OFD1 is more external than the 2 others (Fig 1F and 1G). This localization was further refined by immunogold staining of CEP90a-GFP on whole permeabilized cells in which the gold particles were found mostly at the level of the terminal/intermediate plates, close to the microtubular wall, where transition fibers are known to emerge in *Paramecium* [43] (Fig 1H–1J). Counting of gold particles confirmed that most of the particles are localized at the proximal part of the TZ (65%) (Fig 1J).

### CEP90 depletion prevents basal body docking in *Paramecium*

**Fig 2:**
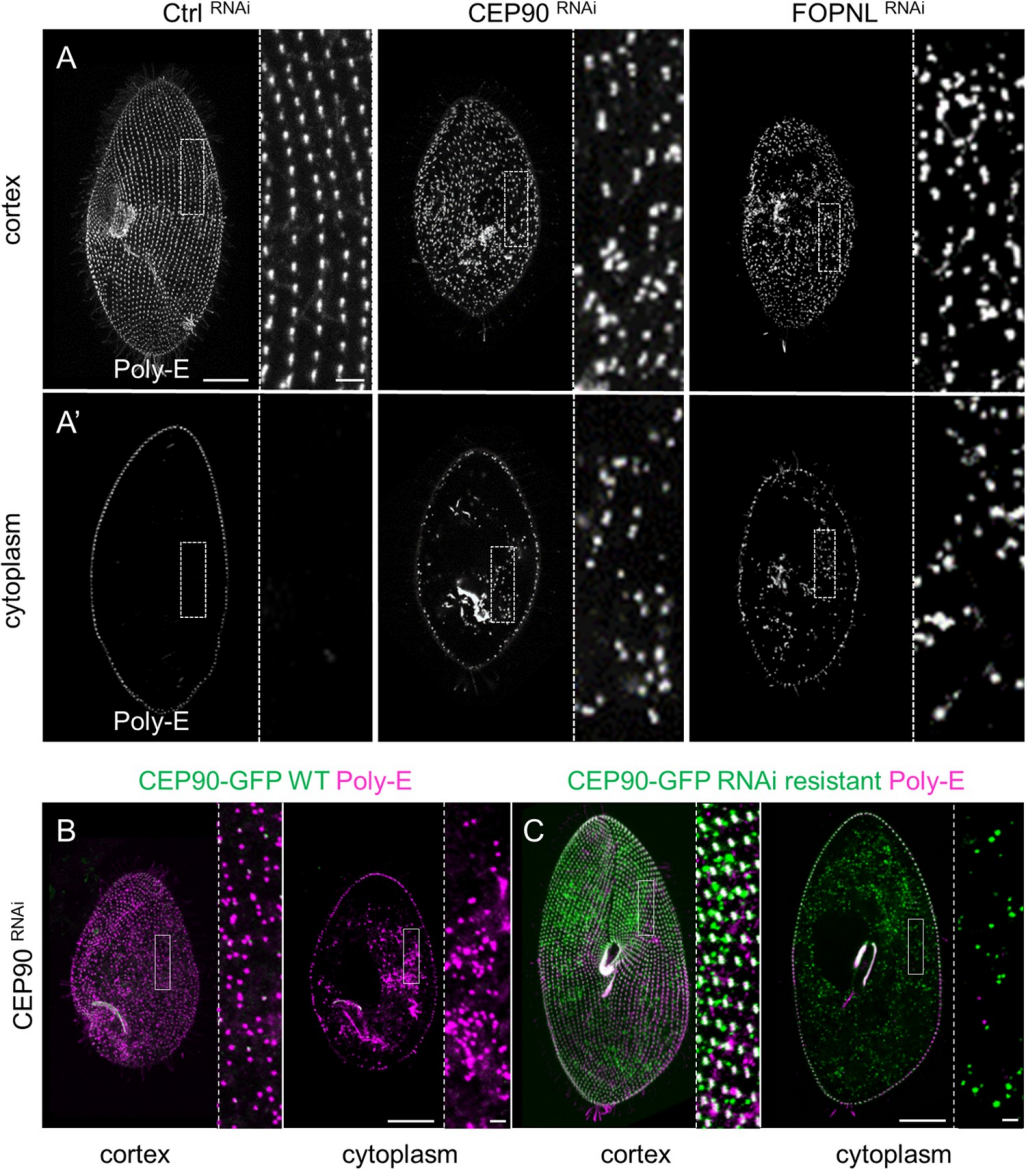
CEP90 depletion affects basal body docking. **(A, A’**) Projection of confocal sections through the ventral surface (**A**) or at the intracytoplasmic level (**A’**), labeled with poly-E antibodies (grey) in control (Ctrl) RNAi, *CEP90*^RNAi^ and *FOPNL*^RNAi^ *Paramecium*. In control-depleted cells, BB are organized in parallel longitudinal rows highlighted in the magnification (right panel) (**A**) with no BB observed in the cytoplasm (**A’**). Cells observed after 2 divisions upon CEP90 or FOPNL depletion appear smaller than control ones, with a severe disorganization of the BB pattern (**A**). Numerous internal BB are observed in the cytoplasm (**A’**). Scale bars = 20 μm and 2 μm (magnification). (**B, C**) Specificity of *CEP90*^RNAi^: transformants expressing either WT CEP90-GFP (**B**) or RNAi-resistant CEP90-GFP (**C**) were observed after 2–3 divisions upon CEP90 depletion and analyzed for green emitted fluorescence and BB staining using poly-E antibodies. Scale bars = 20μm and 2 μm (magnification). The green fluorescence of WT CEP90-GFP transformants was severely reduced along with the expected disorganization of BB pattern at the cell surface (**B**). In contrast, the expression of RNAi-resistant CEP90-GFP rescued endogenous CEP90 depletion and restored the cortical organization of BB (**C**). BB, basal body; RNAi, RNA interference; WT, wild-type.

Since CEP90 was found at proximity of FOPNL by BioID and localized similarly as OFD1-GFP and GFP-FOPNL, we predicted that it will be involved in BB anchoring through its contribution in building the BB distal end. To test this hypothesis, we first decided to knock down *CEP90* in *Paramecium* by feeding wild-type cells with dsRNA-producing bacteria to induce RNAi silencing [44]. As a control, we inactivated the unrelated gene *ND7*, involved in the exocytosis of trichocystes. The high percentage of identity between *CEP90a* and *CEP90b* genes made difficult to design RNAi constructs to inactivate each gene individually; therefore, we silenced them both together. *CEP90* knockdown induced a modification of the cell size and shape from the first division, after what they appeared smaller and rounder (Fig 2A, CEP90^RNAi^) until the third/fourth division, then they start dying (S3B Fig). The swimming velocity was also reduced, as previously observed for defective BB (S3C Fig) [24]. The efficiency of the *CEP90* silencing was tested by immunofluorescence on GFP-CEP90a cells fed by CEP90 RNAi medium to verify the effective depletion (Figs 2B and S3A).

To analyze the effects of the depletion on the BB, inactivated cells were labeled for BB using poly-E antibodies. Whereas control-depleted cells (Ctrl^RNAi^) display BB at the cell surface organized in longitudinal rows (Fig 2A, cortex), CEP90-depleted cells show misaligned BB at the cortical surface (Fig 2A, CEP90^RNAi^). Confocal microscopy demonstrated that numerous BB were found in the cytoplasm after CEP90 depletion, suggesting BB anchoring defects (Fig 2A’, CEP90^RNAi^), while none were observed in control-depleted cells (Fig 2A’, Ctrl^RNAi^). The misaligned BB at the cell surface and cytoplasmic BB observed after CEP90 depletion was reminiscent of the phenotypes observed after depletion of either FOPNL or OFD1 (Fig 2A and 2A’, FOPNL^RNAi^) [29,37]. To ascertain the specificity of this phenotype, RNAi-resistant CEP90a-GFP transformants were inactivated by CEP90 RNAi. GFP staining of RNAi-resistant CEP90a-GFP transformants decorated BB as revealed by poly-E antibodies as CEP90a-GFP expressing paramecia (Fig 2C, cortex). In addition, cytoplasmic aggregates were observed, most probably due to the protein overexpression (Fig 2C, cytoplasm). Importantly, RNAi-resistant CEP90a-GFP expression in paramecia inactivated for endogenous CEP90 rescued the BB organization at the cell surface, which displayed longitudinal rows of BB (Fig 2C). Finally, RNAi-resistant CEP90a-GFP also rescued the dividing time, the survival, and the swimming velocities to the level of control paramecia (S3B and S3C Fig). Altogether, these results suggest that CEP90 is involved in BB anchoring process in *Paramecium*, in agreement with the underlying ciliopathy phenotype observed in human.

**Fig 3:**
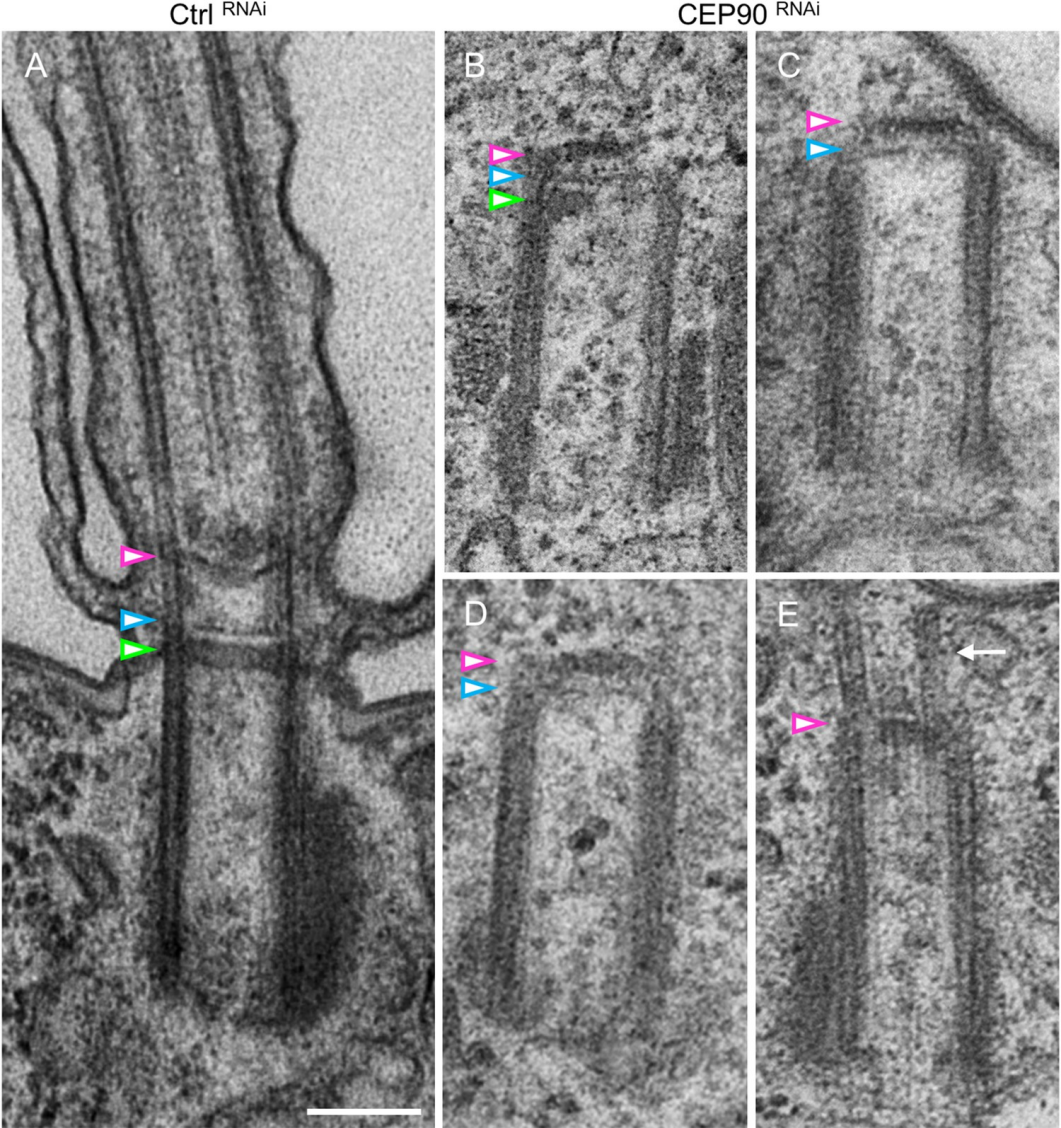
CEP90 depletion affects BB’s distal end maturation. **(A**) *Paramecium* showing 1 ciliated BB after control RNAi depletion (Ctrl). The TZ is characterized by 3 plates indicated by colored arrowheads: terminal plate (green), intermediate plate (blue), and axosomal plate (magenta). (**B-E**) Upon CEP90 depletion, undocked BB are detected close to the cell surface (**C, E**) or deep in the cytoplasm (**B, D**). Vestigial structures resembling the axosomal plate (magenta arrowhead) and intermediate plate (blue arrowhead) are detected on all internal BB (*n* = 63). (**E**) Example of rare BB displaying microtubule extensions on the free, uncapped side of the BB (white arrow). Scale bar = 200 nm. BB, basal body; RNAi, RNA interference; TZ, transition zone.

Next, we used EM to analyze the ultrastructure of the undocked BB and better characterize the BB anchoring defect upon CEP90 depletion. Control-depleted cells show BB anchored at the cell surface and displaying the characteristic 3 plates of the TZ (Fig 3A, arrowheads). In contrast, numerous BB were lying in the cytoplasm in CEP90-depleted cells. All of them display defective distal ends with vestigial structures resembling the intermediate (blue arrowhead) and axosomal (magenta arrowhead) plates, while the terminal plate (green arrowhead) was mostly absent (Fig 3B–3E). Occasionally, microtubule extensions were observed on the uncapped side of the BB (Fig 3E, white arrow).

Since, in mammalian cells, CEP90 was reported to affect centrosome duplication [45], we investigated whether CEP90 depletion could also affect BB duplication in *Paramecium*. To do so, CEP90-depleted cells resulting from the first division upon inactivation were stained for BB. In these cells, the presence of disorganized pattern of BB at the surface attests for an efficient CEP90^RNAi^ during this first duplication. We examine precisely the invariant field localized at the anterior part of these daughter cells, which is characterized by doublet BB units in the wild-type cells. In this field, a defective BB duplication during division leads to singlet BB. As shown on S4 Fig, the invariant field encircled in yellow is mostly constituted of doublets BB in the absence of CEP90. Altogether, these results suggest that in *Paramecium*, CEP90 depletion does not impair significantly the BB duplication process in contrast to its proposed role in mammalian cells [45].

Taken together, these results indicate that CEP90 in *Paramecium* is required to build the distal ends of BB, which is necessary to dock the BB at the cell surface, as already observed for both FOPNL and OFD1.

### CEP90, FOPNL, and OFD1 are required for basal body distal end assembly in *Paramecium*

**Fig 4:**
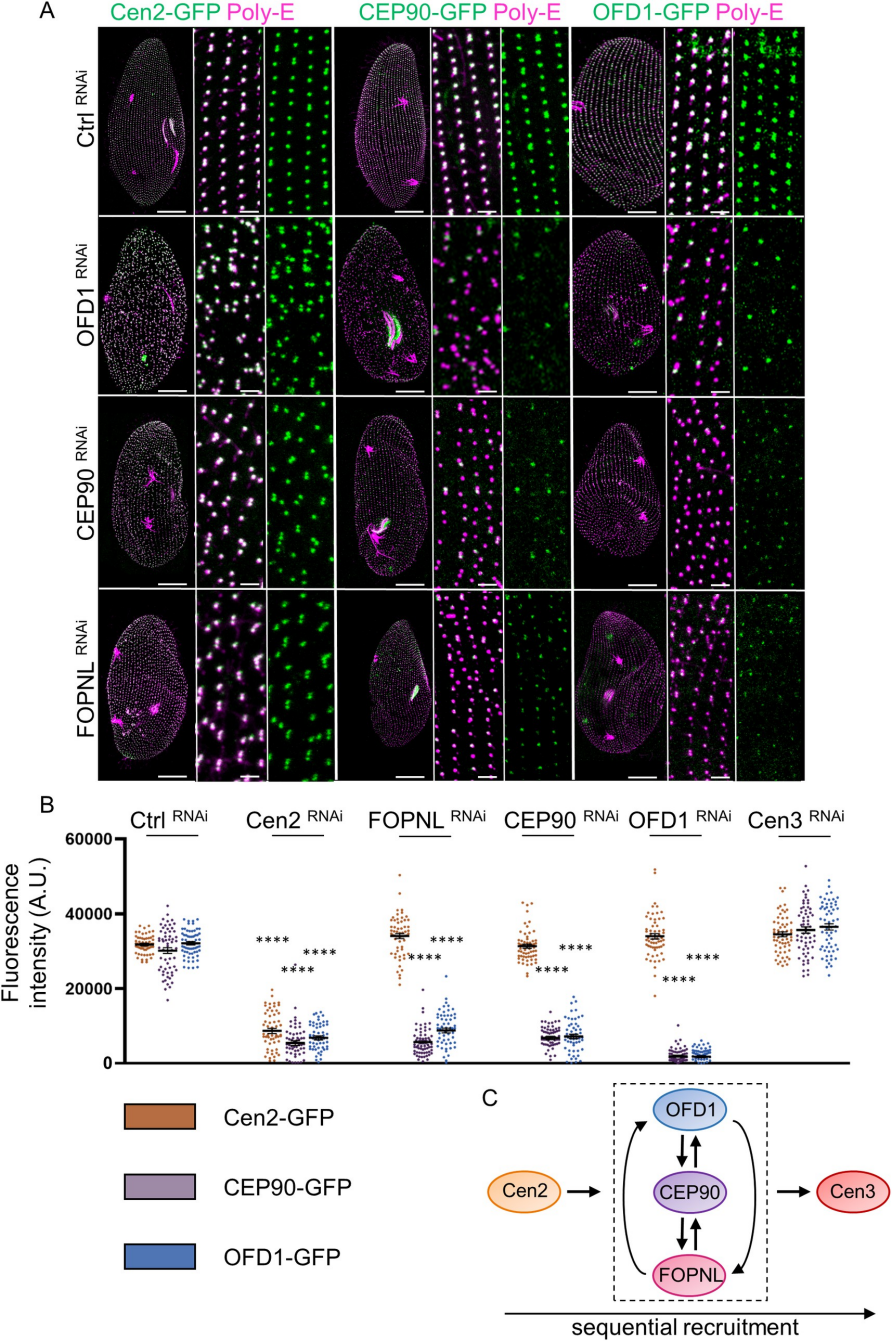
Sequential recruitment of basal body anchoring proteins in *Paramecium*. **(A**) *Paramecium* transformants expressing either Cen2-GFP, CEP90-GFP, or OFD1-GFP (green) and stained for BB (poly-E antibodies, magenta) after 2 or 3 divisions in control cells (Ctrl) and upon OFD1, CEP90, or FOPNL depletion. Control-depleted paramecia displayed well-anchored BB organized in parallel longitudinal rows, with the GFP-emitted signal overlapping BB labeling. After OFD1, CEP90, and FOPNL depletion, BB pattern disorganization is observed. Scale bars = 20 μm and 2 μm (magnification). (**B**) Quantification of the GFP fluorescence of Cen2-GFP (orange), CEP90-GFP (purple), Cen2-GFP (orange), and OFD1-GFP (blue) on newly formed BB in control and upon Cen2, OFD1, FOPNL, CEP90, and Cen3 depletions. The dot plots show the average intensity of GFP fluorescence (*n* > 50 cells, 3 independent replicates). Error bars show SEM. Statistical significance was assessed by one-way ANOVA followed by Tukey’s post hoc test ****p < 0.0001. Source data can be found in S2 Data. (**C**) Schematic representation of the sequential recruitment of BB anchoring proteins leading to proper maturation of BB distal end. Cen2 arrives first at the BB followed by the interdependent recruitment of FOPNL, CEP90, and OFD1. This functional complex is required to allow Cen3 recruitment and BB docking. AU, arbitrary units; BB, basal body.

In *Paramecium*, OFD1 and Centrin2 are required to assemble the BB distal end, as FOPNL [29,37,46]. Centrin2 is recruited first and initiates the assembly of the 3 plates of the BB distal end. This step is followed by a corecruitment of both FOPNL and OFD1 [29,37]. Finally, Centrin3 is recruited to allow the BB tilting up after duplication necessary for its anchoring [46,47]. To determine the involvement of CEP90 in distal end assembly, paramecia expressing GFP-tagged proteins (Centrin2, CEP90a, OFD1, or FOPNL) were inactivated by either Centrin2, OFD1, FOPNL, CEP90, or Centrin3 RNAi. The fluorescence intensity in each transformed cells was quantified by immunostaining after 2 to 3 divisions under RNAi conditions (Fig 4A and 4B). We found that the recruitment of CEP90a-GFP to BB required the presence of FOPNL and OFD1 (Fig 4A and 4B). Reciprocally, CEP90 depletion prevented the recruitment of both OFD1-GFP (Fig 4A and 4B) and GFP-FOPNL (S5A and S5B Fig). These last results suggest an interdependent recruitment of OFD1, FOPNL, and CEP90 to BB. In addition, cells depleted for FOPNL, OFD1, or CEP90 maintained the recruitment of Centrin2 on newly formed BB (Fig 4A and 4B). Moreover, we demonstrated that Centrin2 depletion prevented the recruitment of CEP90 and OFD1 to the newly formed BB (Figs 4B and S5C). By contrast, the depletion of Centrin3, which is not involved in the distal end assembly, did not affect the recruitment of Centrin2-GFP, CEP90a-GFP, and OFD1-GFP (Figs 4B and S5D). Therefore, CEP90, FOPNL, and OFD1 are corecruited at the BB to assemble its distal end in *Paramecium*.

Altogether, these results show (i) the similar localization of CEP90, FOPNL, and OFD1 at BB distal extremity observed by U-ExM; (ii) the interdependent recruitment of these 3 proteins; and (iii) their shared function in BB distal end assembly. This led us to propose that CEP90, FOPNL, and OFD1 could form a functional complex involved in BB distal end maturation and docking.

### Mammalian CEP90, OFD1 localize at the external surface of the distal end of mother and daughter centrioles as well as procentrioles

**Fig 5:**
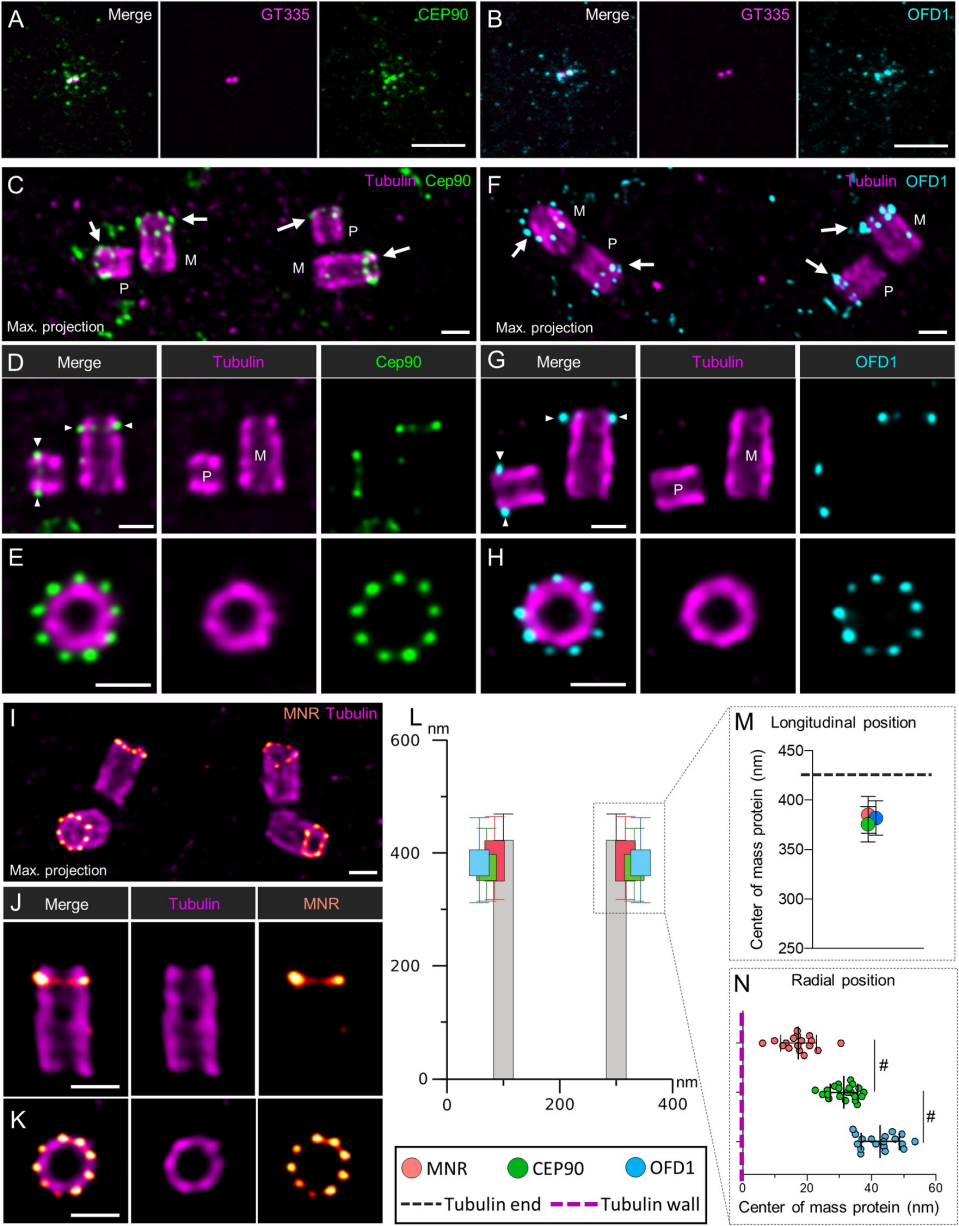
Centrosomal localization of CEP90 and OFD1 in mammalian cells. (**A, B**) RPE1 cells were stained with anti-GT335 antibody directed against tubulin polyglutamylation (magenta) and anti-CEP90 (green) (**A**) or anti-OFD1 (cyan) (**B**). Both CEP90 and OFD1 decorated the centrosome and the centriolar satellites. Scale bar = 5 μm. (**C-J**) U2OS cells were expanded using the U-ExM technique and stained for tubulin (magenta) and CEP90 (green, **C-E**), OFD1 (cyan, **F-H**), or MNR (orange/red, **I-J**). Maximum intensity projections show the organization of CEP90 (**C**), OFD1 (**F**), and MNR (**I**) at the level of the centrosome in duplicating cells (M: Mature centriole; P: Procentriole). Single plan images show that CEP90 (**D**), OFD1 (**G**), and MNR (**J**) localize slightly underneath the distal end of both the mother (M) as well as the procentrioles (P). Finally, images of top-viewed oriented centrioles show 9-fold symmetry organization of CEP90 (**E**), OFD1 (**H**), and MNR (**K**) at the distal end of the centriole. Scale bars = 200 nm. (**L, M**) Distance between the proximal part of centriole (tubulin) and the fluorescence center of mass of CEP90 (green), OFD1 (cyan), or MNR (orange) (longitudinal position). *N* = 23, 25, and 49 centrioles for CEP90, OFD1, and MNR, respectively, from 3 independent experiments. Average +/− SD: CEP90 = 375.3 +/− 17.8 nm, OFD1 = 381.4 +/− 17.4 nm, MNR = 385.1 +/− 18.7. (**L, N**) Localization of CEP90, OFD1, and MNR with respect to the microtubule wall (radial position). Note that MNR localized the closest at the external surface of the microtubule wall, while OFD1 localizes more externally than CEP90. *N* = 21, 18, and 17 centrioles for CEP90, OFD1, and MNR, respectively, from 3 independent experiments. Average +/− SD: MNR = 17.4.1 +/− 5.5; CEP90 = 31.4 +/− 4.3 nm, OFD1 = 42.7 +/− 5.9 nm. Statistical significance was assessed by Kruskal–Wallis test followed by Dunn’s post hoc test, CEP90 vs. MNR *p* = 0.06 (#), CEP90 vs. OFD1 *p* = 0.07 (#). Source data can be found in S3 Data. MNR, Moonraker; U-ExM, ultrastructure expansion microscopy.

To investigate the functional conservation of CEP90 throughout the evolution, we analyzed CEP90 function in mammalian cells. In RPE1 cells, CEP90 localizes at centrioles and centriolar satellites, as previously shown by [45] reminiscent of FOPNL and OFD1 (Figs 5A, 5B and S6A) [19,26]. To further understand the organization of CEP90 in mammalian cells, we used U-ExM to resolve its localization at nanoscale resolution both in the osteosarcoma U2OS and RPE1 cell lines. We observed that, in cycling cells, the endogenous CEP90 or OFD1 longitudinal fluorescence signal is restricted to the centriolar distal end as compared to the tubulin signal, which depicts the whole centriolar length (Figs 5C, 5D, 5F, 5G, S6B and S6C). The precise measurement of both CEP90 and OFD1 staining revealed that these 2 proteins localize on the 2 parental centrioles at about 375 nm from their proximal centriolar extremity (Fig 5C 5F, 5L and 5M) and 50 nm from their distal end of the centrioles. From top-viewed centrioles, we observed a staining organized in a 9-fold symmetry close to the external surface of the centriolar microtubule wall (Fig 5E and 5H). Since OFD1 and CEP90 antibodies were raised in rabbits, double labelling experiments could not be performed. Therefore, the measurements of both CEP90 and OFD1 staining relative to the tubulin maximal intensity signal show that CEP90 is slightly closer to the microtubule wall than OFD1 (average distance between CEP90 and tubulin about 30 nm, while OFD1 is about 40 nm) (Fig 5L–5N). Unfortunately, we have not been able to localize FOPNL using U-ExM, since our antibodies were not working under these conditions. Importantly, the analysis of both CEP90 and OFD1 staining during centriole duplication showed that both proteins were recruited on procentrioles (Fig 5C, 5D, 5F and 5G). Similar results were obtained in RPE1 cells (S6D–S6F Fig). In addition, both ciliated and nonciliated RPE1 BB display an identical CEP90 staining (S6G Fig). Altogether, these results obtained by U-ExM demonstrate that the nanometric localization of both CEP90 and OFD1 in mammalian cells is similar to the one observed in *Paramecium*. The conserved localization between 2 evolutionary distant organisms might suggest a common function. We also investigated the localization of MNR, a direct interactor of OFD1 and FOPNL in mammalian cells [21]. As expected, we found that MNR localizes at the distal end of the parental centrioles on the external surface of the microtubule wall in a 9-fold symmetry similarly as OFD1 and CEP90 (Fig 5I–5K). This new result is in agreement with the relative position between OFD1, CEP90, and NMR [32] but precisely unveils, for the first time, their position relative to the microtubule wall. Quantifications demonstrate that MNR localized in close proximity to the external part of the microtubule wall, next to CEP90 and OFD1, being slightly more external (Fig 5L–5N). Observations in duplicating cells show the recruitment of MNR on early born procentrioles as observed for CEP90 and OFD1 (S6B and S6C Fig).

### The corecruitment of CEP90, FOPNL, and OFD1 is required for the formation of distal appendages

**Fig 6:**
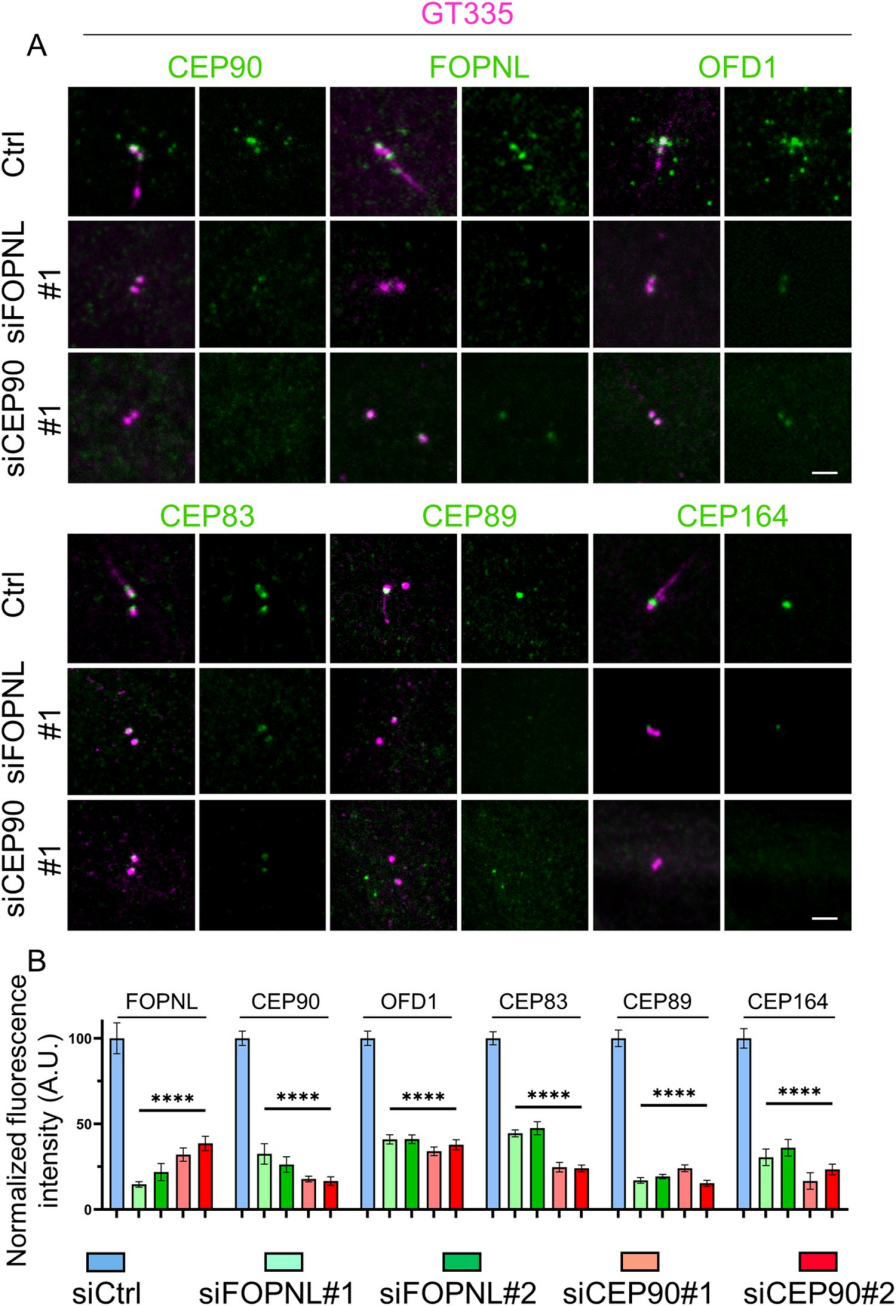
FOPNL and CEP90 allow distal appendage proteins recruitment in mammalian cells. (**A**) Serum-starved RPE1 cells treated with control (ctrl), FOPNL, or CEP90 siRNA were fixed and stained for GT335 (magenta) and antibodies directed against CEP90, FOPNL, OFD1, or the distal appendage proteins CEP83, CEP89, and CEP164 (green). Scale bar = 5 μm. (**B**) Quantification of the fluorescence intensity of FOPNL, CEP90, OFD1, CEP83, CEP89, and CEP164 at the centrosome after control, FOPNL, and CEP90 depletion. All data are normalized base on control siRNA and are presented as average ± SEM. ****p < 0.001 (one-way ANOVA followed by Tukey’s post hoc test), *n* ≥ 50 centrosomes from 2/3 independent experiments. Source data can be found in S4 Data. AU, arbitrary units; siRNA, small interfering RNA.

To investigate the hierarchy of recruitment of these 3 proteins to the centriole distal end in RPE1 cells, we first inactivated either *CEP90* or *FOPNL* gene using 2 sets of siRNA for each gene and found a significant reduction in CEP90 (80%) and FOPNL (80%) levels, respectively, at the centrosome (Figs 6A, 6B and S7A) demonstrating the efficiency of the siRNA, as previously reported [26,45]. Secondly, we analyzed the recruitment of CEP90, FOPNL, and OFD1 to the centrioles upon depletion of CEP90 or FOPNL. We demonstrated that the recruitment of endogenous FOPNL and OFD1 at centrosomes was decreased of 65% upon CEP90 depletion (Fig 6B). Similarly, the depletion of FOPNL decreases the localization of both CEP90 and OFD1 to centrioles of about 70% and 60%, respectively (Fig 6A and 6B). As a control of this experiment, we depleted either MNR or OFD1 by siRNA. In agreement with previous published data, depletion of either MNR or OFD1 in RPE1 cells prevents the recruitment of MNR, FOPNL, and OFD1 [21] (S7A’ and S7B Fig). In addition, both MNR and OFD1 depletion prevents also the recruitment of CEP90 (S7A’ and S7B Fig) [32]. These results suggest that the interdependent recruitment of CEP90, FOPNL, and OFD1 to centrioles and BB is conserved in *Paramecium* and mammalian cells.

To gain further insights into the role of CEP90, OFD1, and FOPNL in ciliogenesis, we depleted either CEP90 or FOPNL proteins in serum-starved RPE1 cells. As expected from previous results [21,26,48], we observed a significant decrease in cilia formation (about 80% compared to control siRNA) (S8A, S8A’ and S8B Fig) in both CEP90 and FOPNL siRNA-treated cells.

Although it was clearly demonstrated that FOPNL controls the assembly of the BB distal end in *Paramecium* together with CEP90 and OFD1, no information concerning the involvement of FOPNL in the distal appendage assembly in mammalian cells was available. Distal appendage formation requires both the presence of specific proteins able to recruit them and the removal of daughter-specific proteins such as Centrobin [49]. First, we analyzed if the depletion of CEP90 or FOPNL may affect the behavior of 1 daughter centriolar protein, Centrobin. As shown in S8C Fig, we did not observe any difference in the localization of Centrobin in control- and FOPNL-depleted cells. Second, we investigated the localization of several distal appendage protein: CEP83, together with CEP89 and CEP164. Consistently, we found that the depletion of FOPNL decreased the localization at the mother centriole of these distal appendage proteins of about 66% (Fig 6A and 6B). Altogether, these results suggest that the depletion of FOPNL prevents the formation of the distal appendages, allowing the understanding of the defective ciliogenesis process previously observed [26,27,30,31]. This suggests that FOPNL is a novel and important component for distal appendage assembly. As expected, depletion of OFD1 and CEP90 prevents distal appendage formation as observed by the poor recruitment of CEP83 and CEP164 as previously shown (S7B Fig) [18]. Similar results were obtained for MNR depletion (S7B Fig) [32]. Consequently, BB docking cannot occur, leading to defective ciliogenesis.

### Overexpressed CEP90 and MNR recruit endogenous CEP83, CEP89, and CEP164 to microtubules

**Fig 7:**
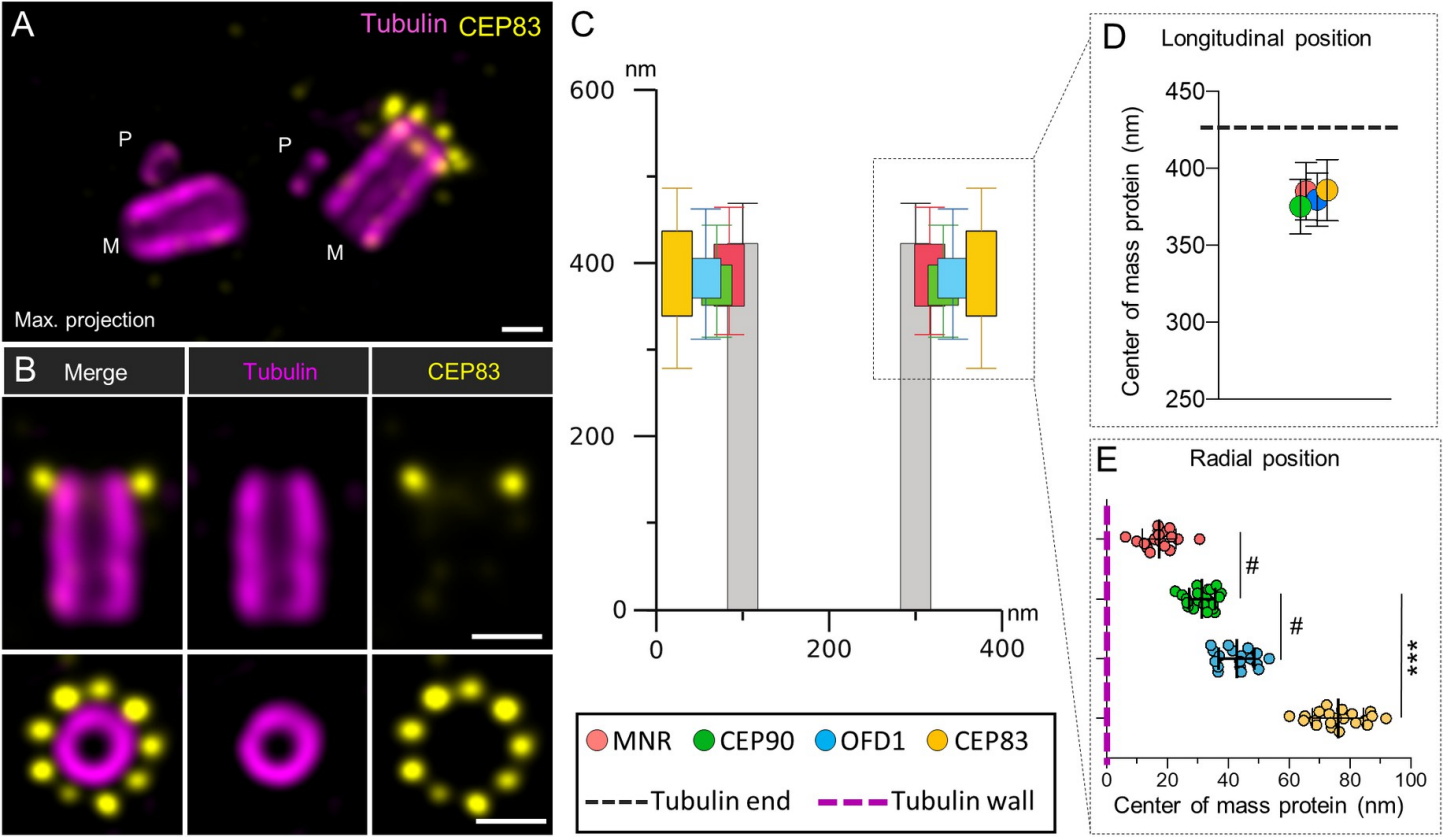
U-ExM localization of CEP83. **(A, B**) U2OS cells were expanded using the U-ExM technique and stained for tubulin (magenta) and CEP83 (yellow). Maximum intensity projection shows the organization of CEP83, which is only present at the distal end of one of the 2 mature centrioles and not at the level of the procentriole (M: Mature centriole; P: Procentriole) (**A**). Single plan images show that CEP83 localizes slightly underneath the distal end of both the mature centriole and is organized in a 9-fold symmetry (**B**). Scale bars = 200 nm. **(C, D**) Distance between the proximal part of centriole (tubulin) and the fluorescence center of mass of CEP90 (green), OFD1 (cyan), MNR (orange), or CEP83 (yellow) (longitudinal position). *N* = 23, 25, 49, and 28 centrioles for CEP90, OFD1, MNR, and CEP83, respectively, from 3 independent experiments. Average +/− SD: CEP90 = 375.3 +/− 17.8 nm, OFD1 = 381.4 +/− 17.4 nm, MNR = 385.1 +/− 18.7 nm, CEP83 = 386.7 +/− 19.9 nm. **(C, E**) Localization of CEP90, OFD1, MNR, and CEP83 with respect to the microtubule wall (radial position). *N* = 21, 18, 17, and 21 centrioles for CEP90, OFD1, MNR, and CEP83, respectively, from 3 independent experiments. Average +/− SD: MNR = 17.4.1 +/− 5.5; CEP90 = 31.4 +/− 4.3 nm, OFD1 = 42.7 +/− 5.9 nm, CEP83 = 76.0 +/− 8.3 nm. Statistical significance was assessed by Kruskal–Wallis test followed by Dunn’s post hoc test, CEP90 vs. MNR *p* = 0.06 (#), CEP90 vs. OFD1 *p* = 0.07 (#), CEP90 vs. CEP83 *p* < 0.0001. Source data can be found in S5 Data. MNR, Moonraker; U-ExM, ultrastructure expansion microscopy.

Our results obtained in mammalian cells showed that (i) the complex composed of FOPNL, OFD1, and CEP90 is required for distal appendage formation; (ii) the nanometric localization of OFD1 and CEP90 is compatible with the localization of CEP83 along the BB proximo-distal axis [50]; and (iii) CEP90, OFD1, and MNR are recruited on early duplicating centrioles. Altogether, these results led us to propose the hypothesis that the FOPNL, OFD1, CEP90 complex will specify, from the procentriole stage, the future position of distal appendages, which will be recruited on the daughter centriole during its maturation into a mother centriole. To test this hypothesis, we first analyzed the localization of CEP83 in U2OS cell lines by U-ExM. As expected, CEP83 is organized in a 9-fold symmetry at a similar position along the centriolar length as CEP90 and OFD1 (Fig 7A and 7B). Quantification of the average diameters of the rings revealed that CEP83 localized slightly more externally than OFD1 and CEP90 (Fig 7C and 7D). These results further support the hypothesis that FOPNL, OFD1, and CEP90 may recruit distal appendage proteins to the centriolar wall.

**Fig 8:**
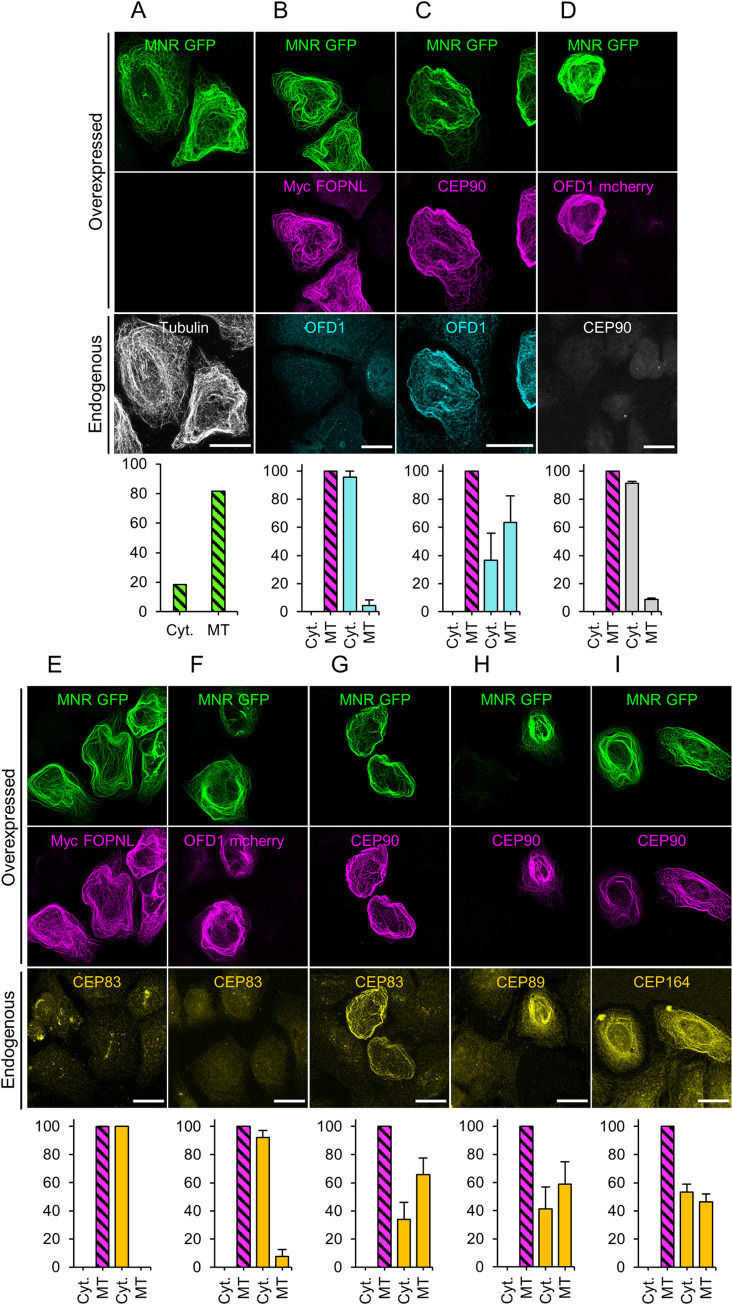
Recruitment of endogenous CEP83 on microtubules by overexpressed MNR-GFP and CEP90. **(A**) U2OS cells transfected with MNR-GFP alone, MNR-GFP and Myc-FOPNL (**B, E**); MNR-GFP and CEP90 (**C, G**); and MNR-GFP and OFD1-mCherry (**D, F**). Transfected cells were stained with the following antibodies to visualize endogeneous proteins: anti-tubulin (**A**), OFD1 (**B, C**), CEP90 (**D**) and CEP83 (**E-G**), CEP89 **(H),** and CEP164 **(I)**. Scale bar = 20 μm. Source data can be found in S6 Data. Cells coexpressed with either GFP-MNR/Myc-FOPNL, GFP-MNR/CEP90, or GFP-MNR/mCherry OFD1 were analyzed for the localization of the endogenous OFD1 (**B**, **C**), CEP90 (**D**), CEP83 (**E**-**G**), CEP89 (**H**), and CEP164 (**I**) proteins. The percentage of co-overexpressed protein localized to microtubules corresponds to 100% (striated bar) and the percentage of the endogenous proteins localized to Cyt. or MTs for each condition. Blue bars correspond to endogenous OFD1; grey bars to CEP90 and yellow bars to CEP83. Averages and SDs are as follows: (**A**) Cyt.: 18.3%; MT: 81.7%, (**B**) Cyt.: 95.6% ± 4.1 MT: 4.4% ± 4.1, (**C**) Cyt.: 36,7% ± 19; MT: 63.3% ± 19, (**D**) Cyt.: 91.3% ± 1.2; MT: 8.7% ± 1.2, (**E**) Cyt.: 100% ± 0; MT:0% ± 0, (**F**) Cyt.: 92.3% ± 4.9; MT: 7.7% ± 4.9, (**G)** Cyt.: 33.3% ± 18.3; MT: 66.7 ± 18.3. **(H)** Cyt.: 41.1% ± 15.8; MT: 58.9 ± 15.8, **(I)** Cyt.: 53.5% ± 5.5; MT: 46.5 ± 5.5. 100 < N < 150 cells per condition from 3 independent experiments. Source data can be found in S6 Data. Cyt., cytosol; MT, microtubule.

In order to decipher which protein of the complex recruits the most proximal distal appendage protein, CEP83, we took advantage of a displacement assay, in which overexpressed MNR-GFP localizes at microtubules and is used as a bait to displace other interacting proteins [21,32]. Therefore, MNR-GFP was overexpressed in U2OS alone or in combination with either myc-FOPNL, mcherry-OFD1, or CEP90. In each transfection condition, the putative displacement of endogenous OFD1, CEP90, MNR, and CEP83 was first analyzed. As expected, expressed MNR-GFP decorated microtubules in U2OS cells (Fig 8A). Unfortunately, we could not displace any endogenous proteins with MNR as bait (S9A Fig). Reasoning that the amount of these proteins might be limited, we next monitored the displacement of overexpressed proteins by MNR-GFP. We demonstrated that MNR-GFP acts as a scaffold to recruit independently overexpressed OFD1, FOPNL, and CEP90 (Figs 8B–8G, S9C, S9E and S9G). Importantly, endogenous OFD1 and CEP83 could be displaced on microtubules of the vast majority of the cell coexpressing CEP90 and MNR-GFP (Fig 8C and 8F). In contrast, only 10% of cells cotransfected with MNR-GFP and OFD1-mcherry were recruiting endogenous CEP90 and CEP83 (Fig 8D and 8G) to microtubules. Since CEP83 is recruited on microtubules in cells coexpressing MNR-GFP and CEP90, we analyzed the localization of 2 other distal appendage proteins localized more distally than CEP83, i.e., CEP89 and CEP164 at the endogenous level. As shown on Fig 8H and 8I, about 53% and 46% of cells expressing both MNR-GFP and CEP90 show a faint relocalization of CEP89 and CEP164, respectively, on microtubules, despite the labeling appearing slightly more diffuse than CEP83. Such a staining is never observed in cells transfected with only MNR-GFP (S9A Fig).

Altogether, these results suggest that MNR interacts, as previously shown, with OFD1 and CEP90 but also with FOPNL. In addition, we discovered that CEP90 recruits 3 distal appendage proteins unveiling that the distal appendage proteins binding to the centriole occurs through CEP90.

## Discussion

In this study, using 2 complementary models, *Paramecium* and mammalian cells, we characterized the evolutionary conserved function of the FOPNL/OFD1/CEP90 module in the BB anchoring process. We demonstrate that these proteins, together with MNR protein in mammalian cells, localize subdistally in a 9-fold symmetry at the external surface of the microtubule wall to recruit distal appendage protein and to initiate their assembly.

Indeed, the search of FOPNL proximity partners in mammalian cells led us to identify several potential interactors. According to previous results [21,32], we detected MNR/OFIP and CEP 90 in proximity of FOPNL as well as several centriolar satellite proteins such as PCM1, CEP350, Cep131, or Talpid3 [33]. Surprisingly, OFD1 is not significantly found despite the presence of biotinylated peptides.

CEP90 was previously identified as a centrosomal and centriolar satellite protein involved in spindle pole integrity in mitotic cells [48] or in centrosome duplication, by recruiting CEP63 to the centrosome [45]. In contrast CEP90 depletion in *Paramecium* could not recapitulate any defect in BB duplication, as we could always detect BB doublets in the invariant field of CEP90-depleted paramecia. One possible explanation could be that, despite numerous conserved actors [51–55], some proteins, such as CEP63, are not present in *Paramecium*, suggesting that the mechanism of BB duplication might differ from *Paramecium* to mammalian cells. We could also speculate that, since *Paramecium* cells are devoid of centriolar satellites, the centriolar satellite pool of CEP90 in mammals is selectively involved in BB duplication, reinforcing previous results [45].

### CEP90, FOPNL, and OFD1 are required for basal body anchoring

Patients with *CEP90* mutations display Joubert and Jeune syndromes, 2 ciliopathies. This result led [48] to postulate that CEP90 function in ciliogenesis may occur through centriolar satellites since their disruption inhibits the ciliogenesis process. We took advantage of the *Paramecium* model, which is physiologically devoid of centriolar satellites, simplifying the readout of the underlying phenotype of CEP90 depletion, to determine the function of CEP90 during ciliogenesis. We found that CEP90 depletion in *Paramecium* prevents BB distal end formation, as previously shown for FOPNL and OFD1, resulting in unanchored BB remaining in the cytoplasm. Interestingly, the depletion of CEP90 prevents maturation of the distal end of BB at the same step as FOPNL or OFD1 [29,37]. This suggests that CEP90, FOPNL, and OFD1 interact functionally to form a functional module involved in BB distal end assembly and in BB docking process. Our results obtained both in *Paramecium* and mammalian cells provide several arguments that may support this hypothesis. First, co-immunoprecipitation experiments performed in mammalian cells show an interaction between FOPNL, OFD1, and CEP90. Second, expansion microscopy performed both in *Paramecium* and mammalian cells demonstrate that the 3 proteins localize similarly, in close proximity of the external of the microtubule wall in a 9-fold symmetry. Third, the depletion of one of these proteins in *Paramecium* or mammalian cells prevents the recruitment of the 2 others. Finally, overexpressed MNR in mammalian cells recruits FOPNL, OFD1, and CEP90, suggesting that these 4 proteins constitute a functional module.

In mammalian cells, the initial step of BB anchoring requires an interaction between the distal appendages and a cellular membrane [11,56–60]. Remarkably, we found that either FOPNL or CEP90 depletion prevents the recruitment of distal appendage proteins, suggesting that the assembly of distal appendages is strongly impaired in these depleted cells as previously shown for OFD1 [18]. Similar results were recently reported in a study performed in mammalian cells showing the role of the MNR, OFD1, and CEP90 complex in distal appendage assembly [32].

Altogether, these results indicate that FOPNL, CEP90, and OFD1 are required to tether the BB at a membrane both in *Paramecium*, bearing motile cilia, and in mammalian cells. Interestingly, *Paramecium* and mammalian cells use 2 distinct BB anchoring pathways. In *Paramecium*, the BB anchors directly to the cell membrane, whereas in RPE1 cells, cytoplasmic vesicles are recruited to the distal appendages, fuse to form the ciliary vesicle, in which the axoneme elongates, suggesting a functional conservation of this molecular module in these 2 evolutionary distant models. Intriguingly, these proteins are absent in *Drosophila* and *C*. *elegans*. A possible explanation might be that in these species, the mechanism of BB anchoring might differ due to the absence of centriolar distal appendage structures. Interestingly, in *Drosophila*, for instance, the distal appendage proteins CEP89, FBF1, and CEP164 are not required for BB docking and TZ formation, despite their recruitment, in a 9-fold symmetry, during BB anchoring (B. Durand, personal communication).

### CEP90, FOPNL, and OFD1 determine the future position of distal appendage proteins

The examination of the localization of CEP90, FOPNL, and OFD1 by U-ExM in *Paramecium* and mammalian cells showed that they localize at the distal end in a 9-fold symmetry at the external surface of the microtubule wall. Combining U-ExM with EM in *Paramecium*, we could determine that CEP90 is located at the most proximal part of the TZ of all anchored BB at the level of the intermediate plate, where transition fibers are known to emerge from the microtubule doublets [43]. This position is identical on both ciliated and unciliated BB and does not vary during the lengthening of the microtubules, which occurs in the TZ during ciliary growth [23]. This is in contrast with the TZ proteins belonging to either MKS or NPHP complexes or the regulators of TZ assembly CEP290 and RPGRIP1L, which are recruited only at the distal part of the TZ of ciliated *Paramecium* BB [24].

In agreement with the results obtained in *Paramecium*, U-ExM in mammalian cells showed that both the mother and the daughter centrioles were decorated by CEP90, OFD1 antibodies. The localization of CEP90, OFD1, and MNR by U-ExM is observed slightly underneath the distal end of the centriole. In agreement with the results reported by Kumar and colleagues [32], MNR diameter is the smallest one. However, costaining of MNR and tubulin antibodies, which precisely determine the localization of the microtubule wall by U-ExM, shows that MNR localization is external of the microtubule wall, as well as CEP90 and OFD1.

**Fig 9:**
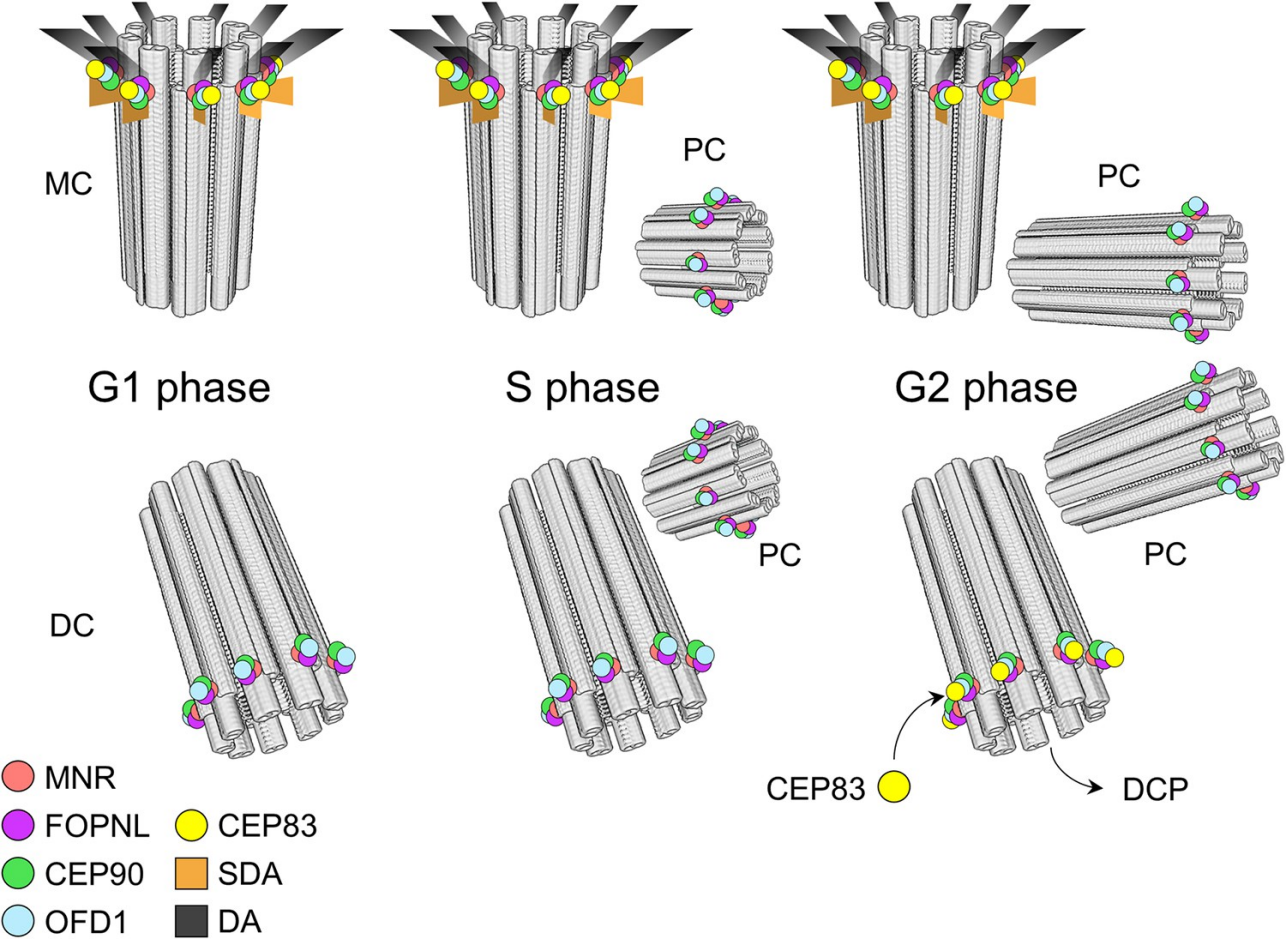
Diagram of the function of the CEP90, FOPNL, and OFD1 in distal appendages assembly. In G1, the complex composed of MNR, CEP90, FOPNL, and OFD1 is localized at both mother (MC) and daughter (DC) centrioles. During centriole duplication (S phase), the complex is recruited on the 2 procentrioles (PC) at the distal position, which is correlated with the future position of distal appendage protein CEP83. During G2/M, the procentrioles elongate maintaining the complex distally. The ancient daughter centriole matures in a mother one, by losing DCP and assembling distal appendages, where the complex is localized. When one of the proteins of this complex (CEP90, FOPNL, and OFD1) is depleted, none of these proteins is recruited to the centriolar wall leading to defective distal end appendage assembly and defective ciliogenesis. We propose that this complex is required to dictate on the procentriole the future location of distal appendage assembly.

The localization of the CEP90, OFD1, and MNR complex is compatible with the localization of CEP83 along the BB proximo-distal axis [50]. Indeed, U-ExM staining of CEP83 in U2OS mammalian cells demonstrates that it was the case. Examination of centrioles during the duplication process in both U2OS and RPE1 cell lines revealed that CEP90 and OFD1 are recruited on the newly born procentriole at a subdistal position similar to the one observed on mature centriole. This result differs from the one observed in [32], which reports the recruitment of the MNR, CEP90, OFD1 complex on fully elongated procentrioles during G2 phase. This discrepancy might be due to the fact that these authors used 5-ethynyl-29-deoxyuridine (EdU) to distinguish the different phases of the cell cycle, while we used unsynchronized U2OS and RPE1 cells. Consistent with our findings, OFD1 has already been observed on newly born procentrioles [18] as well as numerous distal end proteins such as C2CD3, CP110, Centrin2 [20,61,62]. However, the precise localization of these distal end proteins shows some differences: C2CD3 being localized internally of the centriolar microtubule wall, while CP110 is at the extreme distal end. Therefore, the localization of the complex in agreement with the position of the proximal distal appendage protein CEP83 together with the early recruitment on procentriole led us to propose that their presence on the microtubule wall will mark the future position on which distal appendages will assemble. Accordingly, we used a displacement assay based on the overexpression of the microtubule-binding protein MNR to demonstrate that the co-overexpression of MNR and CEP90 is sufficient to recruit ectopically 3 distal appendage proteins, CEP83, CEP89, and CEP164. Based on these results, we propose a model in which MNR binds to the distal end of the centriolar microtubule wall and subsequently recruits OFD1, FOPNL, and CEP90 to that location. Finally, CEP90 recruits CEP83 that will, in turn, recruit the other distal appendage proteins. Altogether, the localization of the complex to procentrioles and the recruitment of endogenous CEP83, CEP89, and CEP164 to co-overexpressed CEP90 and MNR proteins demonstrate that MNR, OFD1, FOPNL, and CEP90 module will recruit distal appendage proteins, leading us to propose that this module will dictate the future position of distal appendages (Fig 9).

However, the reason for the localization of these proteins at the distal ends on both mother and daughter centriole is not clear yet. A possible explanation might come from the results of [49] showing that daughter centriolar proteins (DCPs) are observed on both mother and daughter centrioles after depletion of C2CD3 and Talpid3, which prevents distal appendages assembly, suggesting that DCP are removed from the mother centriole before distal appendages assembly. By contrast, the removal of OFD1 maintained the asymmetric localization of Centrobin on the daughter centrioles [49]. In agreement, the depletion of either CEP90 or FOPNL maintains the asymmetry of the 2 centrioles by maintaining Centrobin only at the daughter centriole. Therefore, we propose the following model: The complex composed of CEP90, FOPNL, and OFD1 is recruited soon after centrosome duplication on the 2 procentrioles, slightly underneath the distal end. POC1B [63] and POC5 [64] are then recruited to allow centriole elongation. Centrin2 [29,46], OFD1 [18], and MNR [32] have been shown to be involved in the regulation of microtubule wall length, since their absence lead to abnormal elongated centrioles. Maturation of the daughter centriole in a mother one is carried out firstly by removing daughter centriole proteins and secondly by the recruitment of distal appendage proteins [49,50,65] during G2/M (Fig 9). We propose that a similar scenario might also been conserved in *Paramecium* since the daughter centriole protein CEP120 is conserved. Intriguingly, according to our previous experiments, MNR is not functionally conserved in *Paramecium*. Therefore, how the functional module FOPNL, OFD1, and CEP90 is tethered to the centriole wall is not clear. In mammalian cells, a functional interaction between C2CD3 and OFD1 has been reported [49,66]. An ortholog of C2CD3 has been found in *Paramecium* [67]. A possibility would be that OFD1 is tethered to the centriolar wall by C2CD3 and will recruit the other member of the module with CEP90 recruiting distal appendages proteins.

To conclude, OFD1, CEP90, and FOPNL appears as a core evolutionary conserved module necessary to allow distal appendage location and assembly both in primary cilia and motile cilia.

## Material and methods

### *P*. *tetraurelia* strains and culture conditions

The wild-type reference strain, stock d4-2 of *P*. *tetraurelia*, was used in RNAi experiment. To facilitate the screening of transformants in microinjection experiments, the mutant *nd7-1* [68], which carries a mutation preventing trichocyst exocytosis was used. Cells were grown at 27°C in wheatgrass infusion (BHB) bacterized with *Klebsiella pneumoniae* and supplemented with 0.8 μg/mL of β-sitosterol according to standard procedures [69].

### Human cell lines and culture conditions

The human retinal pigment epithelium (RPE1), human embryonic kidney (HEK293), human bone osteosarcoma (U2OS) cell lines were grown in DMEM/F12 (RPE1), DMEM (HEK293, U2OS), respectively, supplemented with 10% of fetal bovine serum (FBS) and penicillin-streptomycin (1,000 units/mL and 1,000 μg/mL, respectively HyClone), Glutamax cultured in the presence of 5% CO2. Flp-In T-REx HEK293 cells were grown in DMEM supplemented with 10% FBS, GlutaMAX, zeocin (100 μg/mL), and blasticidin (3 μg/mL). Flp-In T-REx HEK293 stable lines expressing myc-BirA* or myc-BirA*-FOPNL were maintained as above, with the addition of hygromycin (200 μg/mL) instead of zeocin.

### Antibodies

The following antibodies were used: the polyclonal (1/100) and monoclonal (gt335, 1/10,000) PolyE antibody [70] stained cilia; the monoclonal anti-epiplasmin (V3E2) [71]. For mammalian cells, polyclonal rabbit anti-CEP90 (1/250 or 1/500-Proteintech 14413-1-AP; [45]), anti-OFD1 (1/250 or 1/500, Sigma HPA031103; [72]), anti-CEP83 (1/250 or 1/500-Proteintech 26013-1AP; [73]), anti-MNR/KIAA0753 (1/250—Novusbio NBP1-90929) anti-Cep89 (1/800-Proteintech 24002-1AP; [74]), Cep164 (1/1,000-Proteintech 22227-1AP; [50]), anti-Centrobin (1/500-mouse-Abcam-Ab70448; [49], and monoclonal anti-FOPNL antibodies (1/200; [26]) were used. Rat monoclonal anti-MNR were used (1/500; [21]) for microtubule displacement assays while anti-MNR (Novus Biological NBP1-90929) for expansion microscopy. Monobodies AA344 (1:250, scFv-S11B, Beta-tubulin) and AA345 (1:250, scFv-F2C, Alpha-tubulin; [75]) were used in expansion microscopy to stain for the microtubule wall. Goat anti-rabbit Alexa Fluor 488 F(ab’)2 fragment and goat anti-mouse Alexa Fluor 568 IgG (H+L) were provided by Invitrogen and used at 1/1,000.

### BioID sample preparation

Since we searched for proteins at proximity of the centrosomal pool of FOPNL, we first enriched our sample in centrosomes using isolated centrosome-nucleus complexes [76] from HEK293 FlpIn mycBirA*FOPNL and HEK293 FlpIn mycBirA* cells in presence or absence of Tetracycline. BioID purification was then performed according to [77]. Briefly, the purified centrosome-nucleus complexes were then incubated 10 min in 50 mM Tris (pH 7.4), 300 mM NaCl, 0.4% SDS, 5 mM EGTA, 1 mM DTT, Complete anti-protease cocktail (Roche), then 1% Triton-X-100 were added for 10 min. An equivalent volume of Tris (pH 7.4) was added, and the lysate was centrifuged at 4°C at 25,000*g* for 10 min at 4°C. The supernatant was collected and incubated overnight with 70 μL of streptavidin beads with gentle agitation at 4°C. Streptavidin beads were washed with 50 mM Tris (pH 7.4). After the last wash, the proteins were recovered from the beads using Laemmli sample buffer. Proteins were allowed to enter the SDS acrylamide gel for 1 cm. Gels were stained using colloidal blue. The gel containing the proteins is then cut (gel plug). Three independent replicates were done for myc BirA* cells and 2 independent replicates for mycBirA*FOPNL.

### Sample preparation prior to LC-MS/MS analysis

Gel plugs were discolored using a solution of ACN/NH_4_HCO_3_ 50 mM (50/50) during 15 min with agitation. Plugs were reduced with a solution of 10 mM DL-Dithiothreitol (DTT, Sigma-Aldrich, Saint Louis, MO, USA) during 45 min at 56°C, then the plugs were alkylated using a 55-mM solution of iodoacetamide (IAA, Sigma-Aldrich, Saint Louis, MO, USA) during 45 min at room temperature. After a step of washing and dehydration, proteins in the plugs were digested overnight with Trypsin (10 μg/ml) (Promega, Madison, WI, USA) at 37°C in a 25-mM NH_4_HCO_3_ buffer (0.2 μg trypsin in 20 μL). The resulting peptides were desalted using ZipTip μ-C18 Pipette Tips (Pierce Biotechnology, Rockford, IL, USA).

### LC–MS/MS acquisition

Samples were analyzed using an Orbitrap Fusion equipped with an easy spray ion source and coupled to a nano-LC Proxeon 1200 (Thermo Scientific, Waltham, MA, USA). Peptides were loaded with an online preconcentration method and separated by chromatography using a Pepmap-RSLC C18 column (0.75 × 750 mm, 2 μm, 100 Å) (Thermo Scientific), equilibrated at 50°C and operating at a flow rate of 300 nl/min. Peptides were eluted by a gradient of solvent A (H_2_O, 0.1% FA) and solvent B (ACN/H_2_O 80/20, 0.1% FA), the column was first equilibrated 5 min with 95% of A, then B was raised to 28% in 105 min and to 40% in 15 min. Finally, the column was washed with 95% B during 20 min and reequilibrated at 95% A during 10 min. Peptide masses were analyzed in the Orbitrap cell in full ion scan mode, at a resolution of 120,000, a mass range of *m/z* 350 to 1,550 and an AGC target of 4.10^5^. MS/MS were performed in the top speed 3-s mode. Peptides were selected for fragmentation by higher-energy C-trap dissociation (HCD) with a normalized collisional energy of 27% and a dynamic exclusion of 60 s. Fragment masses were measured in an ion trap in the rapid mode, with and an AGC target of 1.10^4^. Monocharged peptides and unassigned charge states were excluded from the MS/MS acquisition. The maximum ion accumulation times were set to 100 ms for MS and 35 ms for MS/MS acquisitions, respectively.

### Data analysis LC-MS/MS

Label-free quantification was done on Progenesis QI for Proteomics (Waters, Milford, MA, USA) in Hi-3 mode for protein abundance calculation. MGF peak files from Progenesis were processed by Proteome Discoverer 2.2 with the Mascot search engine (Matrix Science, version 2.4). Identification of proteins was done with the Swissprot database release 2018_12 with the *Homo sapiens* taxonomy. A maximum of 2 missed cleavages was authorized. Precursor and fragment mass tolerances were set to, respectively, 7 ppm and 0.5 Da. The following posttranslational modifications were included as variable: Acetyl (Protein N-term), Oxidation (M), Phosphorylation (STY), D-Biotin (K). The following posttranslational modifications were included as fixed: Carbamidomethyl (C). Spectra were filtered using a 1% FDR using the percolator node. Protein identifications were only considered when meeting a minimum of 2 unique identified peptides within a single protein. Multivariate statistics on protein measurements were performed using Qlucore Omics Explorer 3.7 (Qlucore AB, Lund, *SWEDEN*). A positive threshold value of 1 was specified to enable a log2 transformation of abundance data for normalization, i.e., all abundance data values below the threshold will be replaced by 1 before transformation. The transformed data were finally used for statistical analysis, i.e., evaluation of differentially present proteins between 2 groups using a Student bilateral *t* test and assuming equal variance between groups. A *p*-value better than 0.05 was used to filter differential candidates.

The mass spectrometry proteomics data have been deposited to the ProteomeXchange Consortium via the PRIDE [78] partner repository with the dataset identifier PXD028740.

### CEP90 gene identification in *Paramecium*

By BLAST search in Parameciumdb (https://paramecium.i2bc.paris-saclay.fr/), we identified 2 *Paramecium* genes encoding proteins homologous to CEP90: *CEP90a* (PTET.51.1.G0320241) and CEP90b (PTET.51.1.G0320283) [79]. CEP 90a and b protein shares about 20.7% identity with human CEP90 protein. These 2 genes result from the last whole genome duplication, which occurred in *Paramecium*, and share 95.2% identity. RNAseq analysis as well as microarray show that *CEP90a* is 10× more expressed than *CEP90b*.

### Gene cloning

For gene silencing, the more divergent part of the gene was chosen to selectively inactivate either *CEP90a* or *CEP90b*, since the 2 paralogs of *CEP90* in *Paramecium* share a high percentage of identity. The inactivation of both CEP90a and CEP90b was performed using an L4440 vector containing the same *CEP90a* fragment (1,472 to 1,951 from ATG start codon) linked to a new fragment of *CEP90b* (139 to 618 from ATG start codon). Since the 139 to 618 *CEP90b* fragment is localized in a less divergent part, an OFF-Target effect has been identified on *CEP90a* with a hit of 3. Silencing vectors of FOPNL, OFD1, PtCen2, and PtCen3 have already been described previously [29,37,46,47]. This vector allows the synthesis of double-stranded RNA corresponding to the cloned gene from 2 T7 promoters. The possible off-target effect of this sequence was analyzed using the RNAi off-target tool in the *P*. *tetraurelia* genome database (https://paramecium.i2bc.paris-saclay.fr/cgi/tool/rnai_off_target) that searches the genome for stretches of 23 identical nucleotides (the size of *Paramecium* siRNA). The only genomic sequence matching for *CEP90a* and *CEP90b* RNAi fragment were *CEP90a* or *b*, thus ruling out possible off-targeting.

For CEP90-GFP expression, *CEP90a* gene showing a 10× higher expression level than *CEP90b* gene during the vegetative state of *Paramecium*, *CEP90a* gene was chosen for expressing the GFP-tagged fusion protein. A synthetic *CEP90a* gene, resistant to *CEP90a* RNAi, was designed by punctual nucleotide modifications (from base 1,472 to 1,951 from the ATG start codon). Both wild-type and RNAi-resistant constructions were inserted in PPXV-GFP plasmids. As a result, the GFP is fused in the 3′ end of *CEP90a* and RNAi-resistant *CEP90a* gene. The fusion proteins are expressed under the control of the calmodulin promoter.

Human cDNA of FOPNL (provided by O. Rosnet) was amplified by PCR using primers (S2 Table) and cloned into pcDNA5 FRT/TO mycBirA* using the appropriate restriction sites. The plasmids MNR-GFP, OFD1-mcherry [21], as well as CEP90 (clone ID 6140514, Dharmacon horizondiscovery).

### Generation of stable and inducible HEK293 flpIn FOPNL for BioID

Flp-In T-REx HEK293 cells were cotransfected with pOG44 (Flp-recombinase expression vector) and either pcDNA5FRT/TO mycBirA*FOPNL or pcDNA5FRT/TO. Transfections were performed with Lipofectamine2000 according to the manufacturer’s instructions. After transfection, cells were selected with hygromycin (200 μg/mL) and blasticidin (15 μg/mL). HEK293 FlpIn mycBirA*FOPNL and HEK293 FlpIn mycBirA* cells were incubated for 24 h with 50 μM biotin (Sigma-Aldrich) in the presence or absence of 0.1 μg/ml tetracycline.

### *Paramecium* transformation

*Nd7-1* cells were transformed by microinjection into their macronucleus [80]. DNA containing a mixture of the plasmid of interest (*CEP90-GFP*, *CEP90RNAi-resistant GFP*, *OFD1-GFP*, *GFP-FOPNL* at 5 μg/μl) with the DNA directing the expression of the *ND7* gene to complement the *Nd7* mutation. Microinjection was made under an inverted Nikon phase-contrast microscope, using a Narishige micromanipulation device and an Eppendorf air pressure microinjector. Transformants were screened for their ability to discharge their trichocysts and further analyzed for GFP. Clones expressing the fusion protein and showing a growth rate similar to untransformed paramecia were chosen for further analyses.

### Gene silencing in *Paramecium* by feeding

L4440 inactivation vectors were introduced in HT115 DE3 *Escherichia coli* strain to produce T7Pol-driven dsRNA as previously described [44]. Paramecia were fed with these bacteria and refed daily with a fresh feeding medium at 27°C. Control cells were fed with HT115 bacteria carrying the L4440 vector containing the *ND7* gene (control). Phenotypes were analyzed after 48 h of feeding. In these conditions, most of the mRNA of silenced genes are knocked down, therefore leading to the depletion of the proteins.

Effectiveness of RNAi was quantified by analyzing the decrease of BB fluorescence in transformed cell lines expressing the GFP-tagged transgene after silencing. In *Paramecium*, the phenotype of silenced cells is highly reproducible from one experiment to another and from one cell to another. In each experiment paramecia were analyzed in at least 2 or 3 independent replicates.

### Paramecia fixation for immunofluorescence

Fixation and immunofluorescence techniques were performed on cells in suspension. Approximately 50 to 100 cells were collected in the smallest volume possible and were permeabilized in 200 μl PHEM (Pipes 60 mM, Hepes 25 mM, EGTA 10 mM, MgCl2 2 mM, adjusted to pH 6.9) with 1% Triton-X-100 (PHEM-Triton) for 30 s. Cells were fixed for 10 min in 2% PHEM-PFA. Buffer was then aspirated and cells were rinsed 3 times for 10 min in PBS–tween 0.1% to 3% BSA

### Confocal acquisition

Confocal acquisitions were made with a Leica SP8 equipped with a UV diode (line 405) and 3 laser diodes (lines 488, 552, and 635) for excitation and 2 PMT detectors. For U-ExM data, images were collected with an inverted confocal Microscope Leica DMI 6000 CS (for *Paramecium* cells) or an inverted Leica TCS SP8 microscope (for human cells) using a 63 × 1.4 NA oil objective with Lightening mode at max resolution, adaptive as “Strategy” and water as “Mounting medium” to generate deconvolved images. 3D stacks were acquired with 0.12 μm z-intervals and an x, y pixel size of 35 nm.

### Expansion microscopy—U-ExM

For analyzing CEP90 and OFD1 in human centrioles, U-ExM was performed as previously described [81]. The following reagents were used in U-ExM experiments: formaldehyde (FA, 36.5% to 38%, F8775, SIGMA), acrylamide (AA, 40%, A4058, SIGMA), N,N′-methylenbisacrylamide (BIS, 2%, M1533, SIGMA), sodium acrylate (SA, 97% to 99%, 408220, SIGMA), ammonium persulfate (APS, 17874, Thermo Fisher), tetramethylethylendiamine (TEMED, 17919, Thermo Fisher), nuclease-free water (AM9937, Ambion-Thermo Fisher), and poly-D-Lysine (A3890401, Gibco). Briefly, coverslips were incubated in 2% AA + 1.4% FA diluted in PBS for 5 h at 37°C prior to gelation in monomer solution (19% sodium acrylate, 0.1% BIS, 10% acrylamide) supplemented with TEMED and APS (final concentration of 0.5%) for 1 h at 37°C and denaturation for 1 h 30 min at 95°C, gels were stained for 3 h at 37°C with primary antibodies: CEP90, OFD1, CEP83, and MNR were used with a combination of tubulin monobodies (see antibodies section) diluted in PBS-BSA 2%. After 3 PBS-tween 0.1% washes, secondary antibodies were incubated for 2 h 30 min at 37°C. Gels were then washed 3 times in PBS-tween 0.1% and incubated in water overnight to allow expansion.

For *Paramecium* BB expansion microscopy, the following modification has been done: purified cortex was incubated in 1%AA + 0.5%FA diluted in 1XPBS for 5 h at 37°C. The GFP-tagged proteins were stained with Rabbit anti-GFP (1/400 Torrey Pines TP-401) and the combination of tubulin monobodies as above.

### Electron microscopy

For ultrastructural observations, cells were fixed in 1% (v/v) glutaraldehyde and 1% OsO4 (v/v) in 0.05 M cacodylate buffer (pH 7.4) for 30 min. After rinsing, cells were embedded in 2% agarose. Agarose blocks were then dehydrated in graded series of ethanol and propylene oxide and embedded in Epon812 (TAAB, Aldermaston, Berkshire, UK). For preembedding immunolocalization, the immunostaining process was carried out as described for immunofluorescence using gold-coupled instead of fluorochrome-coupled secondary antibodies (gold-labeled anti-rabbit IgG-GAR G10, Aurion) diluted 1/30 for 30 min. Cells were then treated as described above.

All ultrathin sections were contrasted with uranyl acetate and lead citrate. The sections were examined with a Jeol transmission electron microscope1400 (at 120 kV).

### *Paramecium* swimming analysis

Approximately 4 to 8 paramecia were transferred in 10 μl drops of conditioned BHB (bacterized BHB solution, which has been depleted for bacteria after their growth and sterilized) for 15 min before being tracked for 10 s every 0.3 s. We used a Zeiss Steni 2000-C dissecting microscope with a Roper Coolsnap-CF camera and Metamorph software (Universal Imaging). Stacks were analyzed using the Manual tracking tool in ImageJ.

### FOPNL immunoprecipitations

Hela Kyoto TransgeneOmics cells expressing GFP-tagged mouse FOPNL were grown, washed in phosphate buffered saline (PBS), and lysed in 50 mM Tris–HCl (pH 7.5), 150 mM NaCl, 1 mM EDTA, containing 1% NP-40 and 0.25% sodium deoxycholate plus a complete protease inhibitor cocktail (Roche) on ice. Clear lysates were obtained after centrifugation 15 min at 13,000 rpm at 4°C. To immunoprecipitate FOPNL, cell lysate was incubated with agarose beads conjugated with either mouse IgG or mouse anti-GFP antibodies (Roche) for 3 h at 4°C under gentle agitation. Beads were washed 5 times with lysis buffer and finally resuspended in Laemmli sample buffer. Proteins were separated by SDS-PAGE and transferred onto nitrocellulose. Blots were revealed with rabbit anti GFP, rabbit CEP90, and rabbit OFD1 antibodies.

### Mammalian cell fixation and immunofluorescence

RPE1 cells were grown on coverslips, washed in PBS, and fixed in methanol at −20°C for 6 min. Alternatively, after a PBS wash, cells were extracted in a PHEM buffer (45 mM Pipes, 45 mM Hepes, 10 mM EGTA,5 mM MgCl2 adjusted to pH 6.9, and 1 mM PMSF) containing 1% Triton-X-100 and then fixed as described above. Cells were rinsed in PBS containing 0.1% Tween 20. Primary antibodies diluted in PBS containing 3% BSA were added for 1 h at room temperature. Three washes were performed in PBS-Tween, and the Alexa-labeled secondary antibodies were applied (Fisher Scientific). Cells were washed in ethanol and mounted in Citifluor (City University, London, England). Observations were done on a confocal microscope. All figures presented are two-dimensional projections of images collected at all relevant z-axes

### siRNA-mediated silencing in mammalian cells

A total of 30,000 RPE1 cells were plated onto coverslips 24 h prior transfection. For CEP90 depletion, 2 siRNAs were used. One is a smart pool of Dharmacon (horizondiscovery) and the other one corresponds to nucleotides 1,651 to 1,675 and was used in the Kodani’s paper [45]. Similarly, 2 siRNAs were used for FOPNL. One is a smart pool of Dharmacon and the other one the published siRNA sequence [26] as well as a smart pool of Dharmacon. Scrambled siRNA was used as a negative control. Cells were transfected with 20 nM of siRNA using Lipofectamine RNAiMax (Invitrogen). Medium was changed after 48 h posttransfection to induce ciliation, and cells were analyzed 72 h posttransfection.

### Microtubule recruitment assay

Approximately 60% to 70% confluent U2OS cells grown on coverslips were transfected in a 6-well plate using lipofectamine 2000 following the manufacturer’s instructions, with 1 μg of total DNA of the following combinations: MNR-GFP alone, OFD1 mCherry alone, CEP90 alone, myc-FOPNL alone, MNR-GFP with either myc-FOPNL, OFD1 mCherry, or CEP90. Expression of the different fusion proteins was allowed for 24 h. Cells were then fixed for 3 min in −20°C cold MeOH and washed once in PBS before staining with antibodies against OFD1, CEP90, myc, CEP83, CEP89, and CEP164. The GFP and mcherry-emitted fluorescence was observed directly. After 3 washes in PBS Tween 0.1%, coverslips were mounted using glycerol-mounting medium and DABCO 1,4-diazabicyclo (2.2.2) octane (Abcam, ab188804).

### Image analysis

For immunofluorescence, image stacks were processed with ImageJ and Photoshop. Confocal acquisition parameters were kept identical (same laser intensity and same gain and same resolution) for quantification of BB fluorescence intensity in control and RNAi-treated cells. Average pixel fluorescence intensity of the different antibodies was then measured within a defined and constant circular area of the size of the BB (*Paramecium*) or centrosome (RPE1 cells), using the measurement tools of ImageJ.

For U-ExM data acquisition of *Paramecium* cortex, confocal images were acquired with a LEICA TCS SP8x (DMi 6000) equipped with a 63× Apochromat oil-immersion objective (numerical aperture (NA): 1.4), U.V diode laser 405 nm and a white light laser (WLL) AOTF coupled. Excitation at 490 nm and 571 nm of Alexa 488 and Alexa 568, respectively. Images were recorded with 2 hybrid detectors. Acquisitions were made at 512 × 512 pixels and 400 Hz speed and zoom of 8.4. Alexa 488 was acquired with a frame accumulation of 3. Sample sampling was 43 nm in XY and for 3D imaging, stacks were acquired with a z-step of 130 nm. The whole system was driven by LAS X software version 3.5.6. Image manipulation were done with ImageJ (Wayne Rasband, National Institutes of Health). Images were deconvolved employing the Huygens Essential package (Scientific Volume Imaging, Hilversum, the Netherlands), with sampling of 43 nm in XY and 130 nm in Z and the following parameters: algorithm: CMLE; signal/noise ratio: 20; quality threshold: 0.01%; maximum iterations: 40; background calculation: in/near objects.

For U-ExM analysis, length coverage and diameter quantification was performed as previously published in [81].

To generate the panels in Figs 1F, 1G, 5L, 5M, 5N, 7C, 7D and S2F, 2 homemade plugins for ImageJ were used. The first one helps the user to easily pick the extremities of a centriolar protein signal. For each protein of each image, the user picks the first and the last point of the signal along the centriole length. This generates an entry in a table for each centriole describing where the proteins start and end. The results are saved in a.csv file that can be directly read by the second plugin. The second plugin reads the table generated by the first plugin and summarizes the positions of different proteins in one plot. For each centriole, one protein (here the tubulin) is defined as the reference protein and its Y coordinates are shifted so its Y starting point is set to 0. The same shift is applied to the second protein to keep the correct distances between the 2 proteins. The Y coordinates values of both signals are then rescaled to the average length of the reference protein. For each protein, the average and side deviation of the Y starting position and of the Y ending position is calculated. A plot is then generated illustrating the average position of one protein to the other.

### Statistical analysis

The statistical significance of the difference between groups was determined using one-way ANOVA followed by Tukey’s post hoc test. All data are presented as an average +/− SEM as specified in the figure legends. Differences were considered significant when *p* < 0.05. Results reported are from 2 to 3 independent biological replicates as noted in legends with reproducible findings each time.

## Supporting information

S1 Table myc-BirA*-FOPNL BioID raw protein measurement. MS/MS comparison of 2 biological replicates of the myc-BirA*-FOPNL with the myc-BirA*. High-confidence interactors were defined as those with q value <0.05 and max fold >5.
(XLSX)

S2 Table Primer, RNAi, siRNA, and CEP90-GFP RNAi-resistant sequences. All primers used to construct *Paramecium* CEP90-GFP and CEP90-GFP-resistant plasmid as well as *CEP90a* and *CEP90b* gene silencing vectors were synthesized by Eurofins Genomics. The RNAi-resistant sequence of CEP90-GFP RNAi-resistant plasmid (red) was designed following the ciliated codon code and was synthesized by Integrated DNA Technologies. siRNA sequences directed against CEP90 and FOPNL were synthesized by Dharmacon according to the manufacturer’s protocol. In both cases, a published single sequence was used (siRNA CEP90 #2 and siRNA FOPNL #1) as well as a combination of 4 sequences directed against the gene (smartpool). IP, immunoprecipitation; RNAi, RNA interference.
(PDF)

S1 Fig FOPNL, CEP90, and OFD1 form a complex in mammalian cells. (**A)**. VolcanoPlot of proteins identified in FOPNL-myc-BirA* + tet versus myc-BirA*+ tet, made with Qlucore. The X-axis shows the LOG2 of the Fold Change and the Y-axis shows the -LOG10 of the *p*-value derived from a Student bilateral *t* test assuming equal variance between groups. Differential genes satisfying both *p*-value <0.05 and Fold Change > 5 were colored in red. (**B)** Heatmap of proteins identified in myc-BirA*-FOPNL + tet versus myc-BirA*+ tet, made with Qlucore. Proteins were filtered based on a *p*-value < 0.05 and Fold Change > 5, intensities were log_2_ transformed and were displayed as colours ranging from red to blue. Both rows and columns were clustered hierarchically. Source data can be found in S7 Data. (**C)** Protein–protein interaction network from differentially abundant proteins identified in FOPNL-BirA+tet versus BirA*+tet. Proteins were filtered based on a *p*-value <0.05 and Fold Change >5. The network was created using STRING DB representing a full STRING network at medium confidence (0.400) with disconnected nodes hidden. Lines of different thicknesses between nodes symbolize the Edge confidence from medium to the highest. The cluster encircled in purple indicates centriolar and centriolar satellite proteins. The one in green corresponds to nuclear proteins. (**D)** Co-immunoprecipitation experiment made in HeLa Kyoto GFP-FOPNL. Cell extract was immunoprecipitated with mouse control or anti-GFP immunoglobulins. The input, the unbound fraction (FT) as well as the bound fraction (IP) were revealed with either rabbit polyclonal anti-GFP, CEP90, and OFD1 antibodies. Both CEP90 and OFD1 are co-immunoprecipitated by GFP antibodies, indicating a complex between FOPNL, OFD1 and CEP90. tet, tetracyclin.
(TIF)

S2 Fig Localization of CEP90-GFP, OFD1-GFP, and GFP-FOPNL by U-ExM. **(A**) EM on *Paramecium* cortex fragment showing a 2-BB unit. During cortex purification, cilia shed above the TZ (white arrowhead). The unciliated BB (yellow arrowhead) remain unmodified. Scale bar = 100 nm. (**B-E**) U-ExM performed on paramecia transformants expressing CEP90-GFP (**B**), OFD1-GFP (**C, E**), and GFP-FOPNL (**D**) show BB and cilia stained with tubulin antibody (magenta). Note that a space indicated by a white arrowhead shows the cilium breakage between the distal end of the TZ and the cilium. (**B, C**) CEP90-GFP and OFD1-GFP signals are localized at the distal extremity of nonciliated BB (yellow arrowhead), while GFP signal is slightly underneath the distal end of ciliated BB (white arrowhead). Scale bar = 250 nm. (**F**) Schematic representation of OFD1 (blue square), FOPNL (yellow square), and CEP90 (green square) localization on ciliated and unciliated BB. Scale bar = 200 nm. Source data can be found in S8 Data. BB, basal body; TZ, transition zone; U-ExM, ultrastructure expansion microscopy.
(TIF)

S3 Fig CEP90 depletion phenotypes. (**A-C**): *Paramecium* were transformed with a construction expressing either WT CEP90-GFP or an RNAi-resistant CEP90-GFP. (**A**) Quantification of BB GFP signal fluorescence on newly formed BB (anterior ones) after 2 or 3 divisions upon CEP90 depletion. average ± SEM is represented, ****p < 0.001 (one-way ANOVA followed by Tukey’s post hoc test), *n* ≥ 60 BB from 2 independent experiments. Source data can be found in S9 Data. (**B**) Number of cell divisions in control, CEP90, and FOPNL depleted cells as well as in cells rescued with RNAi-resistant CEP90. Note that both CEP90 and FOPNL depleted cells died after 4 or 5 divisions. Source data can be found in S9 Data. (**C**) Quantification of the swimming speeds of noninjected paramecia (NI), paramecia expressing WT CEP90-GFP, or RNAi-resistant CEP90-GFP after 48 h under control condition or CEP90 RNAi condition. Each dot shows the average velocity of 1 cell, average ± SEM is represented, ****p < 0.001 (one-way ANOVA followed by Tukey’s post hoc test, *n* ≥ 60 cells per condition performed from 2 independent experiments). Source data can be found in S9 Data. BB, basal body; RNAi, RNA interference; WT, wild-type.
(TIF)

S4 Fig CEP90 depletion does not affect BB duplication. (**A)** Early stage of *Paramecium* division stained with poly-E antibodies (grey). Cells were observed after control (Ctrl^RNAi^) and CEP90 depletion (CEP90^RNAi^) after the first division. The posterior part of the cell shows defective BB organization in CEP90-depleted cell, suggesting that the RNAi has been effective. The invariant field encircled in yellow on the figure shows the usual 2 BB pattern at the cortical level, suggesting that BB duplication has occurred normally. Defective BB duplication will lead to only 1 BB in this field. Sometimes additional BB are found but appear below the cortical surface (yellow arrowheads). Scale bars = 20 μm. BB, basal body; RNAi, RNA interference.
(TIF)

S5 Fig Recruitment of CEN2-GFP, CEP90-GFP, OFD1-GFP, and GFP-FOPNL in Cen2, Cen3, and CEP90-depleted cells to newly formed BB. (**A**) Transformants expressing a GFP-tagged FOPNL were observed 2–3 divisions upon control (Ctrl) or CEP90 depletion. GFP-FOPNL fluorescence (green) was severely reduced on newly formed BB along with the expected BB pattern disorganization. Scale bar = 20 μm. (**B**) Dot plots showing the average percentage of GFP-FOPNL BB fluorescence after 2–3 divisions in either FOPNL or CEP90 RNAi (*n* ≥ 100 BB per condition performed in 2 independent replicates). Error bars show SEM. Statistical significance was assessed by a one-way ANOVA followed by Tukey’s post hoc test *p* < 0.0001 ****. Source data can be found in S10 Data. (**C, D**) *Paramecium* transformants expressing Cen2-GFP, CEP90 GFP, or OFD1-GFP (green) have been stained for BB (poly-E antibodies, magenta) after 2–3 divisions upon Cen2 **(C)** or Cen3 **(D)** depletion. After Cen2 and Cen3 depletion, BB pattern disorganization is observed. (**C)** The GFP fluorescence is not recruited to newly formed BB after Cen2 RNAi. (**D)** By contrast, Cen3 depletion did not affect neither CEP90-GFP, OFD1-GFP, and GFP-FOPNL recruitment to BB. Scale bars = 20 μm and 2 μm (magnification). AU, arbitrary units; BB, basal body; RNAi, RNA interference.
(TIF)

S6 Fig Localization of FOPNL, CEP90, and MNR. (**A**) HeLa-Kyoto cell lines expressing GFP-FOPNL (green) were stained for CEP90 (magenta). Confocal images show the colocalization of both proteins at centrosome and centriolar satellites. Scale bar = 5 μm. (**B**, **C**) Confocal images of duplicating U2OS cell, expanded and stained for tubulin (magenta) and CEP90 (green), OFD1 (cyan), or MNR (orange/red) showing the recruitment of the 3 proteins at early stages of procentriole assembly, slightly after the arrival of the tubulin (PC: procentriole, MC: mature centriole). Scale bars: 200 nm. (**D-F**) Confocal images of duplicating RPE1 cells, expanded and stained for tubulin (magenta) and CEP90 (green) showing the presence of CEP90 slightly underneath the distal end of both the mother (MC) and procentrioles (PC). Note that CEP90 is recruited at early stages of procentriole assembly just after the start of the tubulin (arrowheads) Scale bar = 200 nm. (**G**) Confocal images of ciliated RPE1 cell, expanded and stained for tubulin (magenta) and CEP90 (green) showing the presence of CEP90 at the distal part of the BB templating the primary cilium (C). Please note that the daughter centriole (DC) is also positive for CEP90 as shown in the upright insert where the distal part of the DC is shown. Scale bar = 200 nm. BB, basal body; DC, daughter centriole; MC, mature centriole; MNR, Moonraker; PC, procentriole.
(TIF)

S7 Fig Localization of CEP90, FOPNL, OFD1, CEP83, CEP89, CEP164 in FOPNL and CEP90-depleted cells. **(A**) Representative images of distal appendage proteins labelling after depletion of CEP90 (siRNA#2) or FOPNL (siRNA#2), stained with CEP90, FOPNL, OFD1, CEP83, CEP89, CEP164 antibodies (green). The centrosome is stained with GT335 (magenta). Control siRNA and CEP90 (siRNA#1) and FOPNL (siRNA#1) are found in Fig 6A. (**B)** Quantification of the fluorescence intensity of antibodies: CEP90, OFD1, MNR, FOPNL, CEP83, and CEP164 staining at the centrosome. All data are presented as average ± SEM. ****p < 0.001 (one-way ANOVA followed by Tukey’s post hoc test), *n* ≥ 100 centrosomes in 3 independent experiments. Source data can be found in S11 Data. AU, arbitrary units.
(TIF)

S8 Fig Effect of CEP90 and FOPNL silencing in primary cilia formation and centrobin localization. **(A, A’**) Serum-starved RPE1 during 24 h in control (siCtrl) condition and upon CEP90 (**A**) or FOPNL (**A’**) depletion were stained for GT335 (magenta) and CEP90 (**A**, green) or FOPNL (**A’**, green). Scale bars: 15 μm. (**B**) Percentage of ciliated cells were quantified in each condition. Average ± SEM is represented, ****p < 0.001 (one-way ANOVA followed by Tukey’s post hoc test, *n* ≥ 350 cells per condition performed in 2 independent replicates). Source data can be found in S12 Data. (**C**) The daughter centriolar protein Centrobin was localized in control (siCtrl), CEP90, and FOPNL-depleted RPE1 cells. No significant differences are observed between control-depleted cells and CEP90 or FOPNL-depleted cells. Scale bar = 5 μm.
(TIF)

S9 Fig Control experiments of the recruitment of endogenous CEP83 on microtubules by MNR-GFP and CEP90 overexpression. **(A)** U2OS cells overexpressing MNR-GFP stained for endogenous OFD1, CEP90, CEP83, CEP89, and CEP164 as well as the percentage of cells displaying endogenous proteins to either Cyt or MT. Scale bars: 20 μm. Source data can be found in S13 Data. (**B, D, F**) Overexpression of myc-FOPNL, CEP90, and OFD1 mcherry and their localization to the cytoplasm as well as the percentage of cells displaying the localization of proteins to either Cyt or MTs. **(C, E, G**) Co-overexpression of myc-FOPNL, CEP90, and OFD1 mcherry with MNR-GFP. Note that the cytoplasmic localization of myc-FOPNL, CEP90, and OFD1 mcherry shift with MNR on the microtubules. Percentage of cells displaying the localization of proteins to either Cyt or MTs. Scale bars = 20μm. Source data can be found in S13 Data. Cyt, cytoplasm; MNR, Moonraker; MT, microtubule.
(TIF)

S1 Data Raw data of the mean distance between the protein of interest and either the proximal end of microtubule wall or the microtubule wall. Data corresponding with Fig 1F and 1G.
(XLSX)

S2 Data Quantification of the GFP fluorescence of Cen2-GFP, CEP90-GFP, Cen2-GFP, and OFD1-GFP on newly formed BB in Control and upon Cen2, OFD1, FOPNL, CEP90, and Cen3 depletions. All raw data are included. Data corresponding with Fig 4B.
(XLSX)

S3 Data Raw data of the mean distance between the protein of interest and either the proximal end of microtubule wall or the microtubule wall. Data corresponding with Fig 5L–5N.
(XLSX)

S4 Data Quantification of the fluorescence intensity of FOPNL, CEP90, OFD1, CEP83, CEP89, and CEP164 at the centrosome after control, FOPNL, and CEP90 siRNA-mediated silencing. All raw data are included. Data corresponding with Fig 6B.
(XLSX)

S5 Data Raw data of the mean distance between the protein of interest and either the proximal end of microtubule wall or the microtubule wall. Data corresponding with Fig 7C–7E.
(XLSX)

S6 Data (A-D) Number of cells transfected with MNR-GFP or cotransfected with MNR-GFP/myc FOPNL, MNR-GFP/CEP90, MNR-GFP/OFD1mcherry showing decorated microtubules (MT) or not (Cyt) using tubulin, OFD1, CEP90 antibodies. (E-I) Number of cells cotransfected with MNR-GFP/myc FOPNL, MNR-GFP/CEP90 showing decorated microtubules (MT) or not (Cyt) with CEP83, CEP89, CEP164. All raw data are included. Data corresponding with Fig 8A–8D and 8E–8I.
(XLSX)

S7 Data (A) Raw data of the proteins identified in myc-BirA*-FOPNL + tet versus myc-BirA*+ tet, made with Qlucore to construct the volcanoplot. (B) Raw data corresponding to the heatmap. (C) Original western blot corresponding with S1D Fig. All raw data are included. Data corresponding with S1A, S1B and S1D Fig.
(XLSX)

S8 Data Raw data of the mean distance between the protein of interest and the proximal end of microtubule wall. Data corresponding with S2F Fig.
(XLSX)

S9 Data (A) Quantification of BB GFP signal fluorescence on newly formed BB (anterior ones) upon CEP90 depletion. (B) Number of cell divisions in control, CEP90, and FOPNL-depleted cells as well as in cells rescued with RNAi-resistant CEP90. (C) Quantification of the swimming speeds of noninjected paramecia or paramecia expressing WT CEP90-GFP or RNAi-resistant CEP90-GFP depleted with control or CEP90 RNAi. All raw data are included. Data corresponding with S3A–S3C Fig.
(XLSX)

S10 Data Quantification of the GFP fluorescence of GFP-FOPNL on newly formed BB in Control and upon FOPNL or CEP90 depletions. All raw data are included. Data corresponding with S5B Fig.
(XLSX)

S11 Data Quantification of CEP90, OFD1, FOPNL, MNR, CEP83, and CEP164 at the centrosome after control, MNR, or OFD1 siRNA-mediated silencing. All raw data are included. Data corresponding with S7B Fig.
(XLSX)

S12 Data Quantification of the number of ciliated cells after control, FOPNL, or CEP90 siRNA-mediated silencing. All raw data are included. Data corresponding with S8B Fig.
(XLSX)

S13 Data (A) Number of cells transfected with FOPNL, CEP90, OFD1 mcherry, or cotransfected with MNR-GFP/myc-FOPNL, MNR-GFP/CEP90, MNR-GFP/OFD1 mcherry showing decorated microtubules (MT) or not (Cyt) using tubulin, OFD1, CEP90, CEP83, CEP89, and CEP164 antibodies. (B-G) Number of cells transfected with FOPNL, CEP90, OFD1 mcherry, or cotransfected with MNR-GFP/myc FOPNL, MNR-GFP/CEP90 displaying the localization of proteins to either cytoplasm (Cyt) or microtubules (MT). All raw data are included. Data corresponding with S9A and S9G Fig.
(XLSX)

## Abbreviations

BB: basal body
Bio-ID: Identification by in vivo biotinylation
DC: daughter centriole
DCP: daughter centriolar protein
Edu: 5-ethynyl-29-deoxyuridine
EM: electron microscopy
IP: immunoprecipitation
KO: knockout
MC: mother centriole
MNR: Moonraker
PC: procentriole
RNAi: RNA interference
siRNA: small interfering RNA
tet: tetracyclin
TZ: transition zone
U-ExM: ultrastructure expansion microscopy
