# Supplementary material for: The evolutionary conserved proteins CEP90, FOPNL, and OFD1 recruit centriolar distal appendage proteins to initiate their assembly
Source: PLoS Biol. 2022 Sep 7;20(9):e3001782. doi: 10.1371/journal.pbio.3001782 (PMC9484695; doi:10.1371/journal.pbio.3001782)
Supplement: S2 Table — All primers used to construct Paramecium CEP90-GFP and CEP90-GFP-resistant plasmid as well as CEP90a and CEP90b gene silencing vectors were synthesized by Eurofins Genomics. The RNAi-resistant sequence of CEP90-GFP RNAi-resistant plasmid (red) was designed following the ciliated codon code and was synthesized by Integrated DNA Technologies. siRNA sequences directed against CEP90 and FOPNL were synthesized by Dharmacon according to the manufacturer’s protocol. In both cases, a published single sequence was used (siRNA CEP90 #2 and siRNA FOPNL #1) as well as a combination of 4 sequences directed against the gene (smartpool). IP, immunoprecipitation; RNAi, RNA interference. (PDF) [file pbio.3001782.s002.pdf]

Table S2	Primers, RNAI, siRNA and CEP90-GFP RNAI resistant sequences	
	**Primer Paramecium**	
**Constructs**	**Primers**	
**CEP90a-GFP**	CEP90a sens	caaatcattaattaaataaatcaactagtATGAGTACGAAGAGAAAAGACATCATAAAAC
	CEP90a anti-sens	CTTCTCCTTTAGACATgcgtacctcgagTCCTCCTCCTTTATTCTGTTTTTGTTTC
**CEP90a-GFP RNAi resistan**	CEP90a-GFP RNAi resistant sens A	GGATAGAGTATAGAATATCTATGAAGATACATTGGCTAATTTGAAAGCCACAAAACATGAAAACG
	CEP90a-GFP RNAi resistant antisens A	CGTTTTCATGTTTTGTGGCTTTCAAATTAGCCAATGTATCTTCATAGATATTCTATACTCTATCC
	CEP90a-GFP RNAi resistant sens B	GGATAAAACCCACTAACCTCACTCCTACCTCGTCGCCCATATTGAGGAAAAGGATAGAGAAATTTTG
	CEP90a-GFP RNAi resistant antisens B	CAAAATTTCTCTATCCTTTTCCTCAATATGGGCGACGAGGTAGGAGTGAGGTTAGTGGGTTTTATCC
**RNAi CEP90**	CEP90a sens	CCATTTAAAAAGGAAGGGGAATTTTAGAAACTTTGGCCAATCTTAAAGCTACAAAGCATG
	CEP90a antisens	CACCGCGGTGGCGGCCGCTCTAGAACTAGTTACGAGATAGGAATGActaaaataaaag
	CEP90b sens	CCATTTAAAAAGGAAGGGGAATTTTAGAAACTTTGGCCAATCTTAAAGCTACAAAGCATG
	CEP90b antisens	CATGCTTTGTAGCTTTAAGATTGGCCAAAGTTTCTAAAATTCCCCTTCCTTTTTAAATGG
	**RNAi *Paramecium***	
**RNAi CEP90a**		CTTTGGCCAATCTTAAAGCTACAAAGCATGAAAATGAAATGTTAAGGGAGAAGATCAACGTGTTGAAGGCAGAGTATTATAAGTGTTAGGTAGAGGCTAAGGAATAGATGAGCAGCATAAATGCTTAATTGCAAGTTGCAAAAGAACAATTAACTAATTATGAAGGAATTGAAAAAGAAATTGATGATGC<br>CATAATGAAATCAGCAGGCAATGAATATGATGCAATGAATCCTTTATTGGGATTCATGGGCACTGTACCTACATCTTCAAAAAGAAGGATTCAACAGGCATTAAATCTCGCTTAGAGATTGCAAGCAAAATAAAGAGAATCAGAGGAATTGCAAAGACAATTAAGAGATAAGTCATAAGAGATTGAGAGA<br>CTATAGGAAGAGACCAAATTATAAAGAGATGTACTTGACAAAACACATTAACCgtaaatattcattaaatatcttttattttagTCATTCCTATCTCGTA
**RNAi CEP90b**		GATTCTGTATCCATAGCTGATATTGAGAATTTTTAATAGAAGAGACATAATAGAGGAGGGGATACTCACGACACAGTCAATATGAAAAATgtattataattgattcaataatagAATTATTCTTCTGTAGTGGGCATGCAAGAGAAATAAATAGCATTTCTAAATGCAGAAATAAAGCGGATTCAAGCAGCTAACGATGCT<br>GATAGACAAAGGATTTTTGCTAATCATAGAATTGAAACTGATAAGCTTTTAAGTGAAATCAAACAACTTAAGTCTAAGATGATATATTAAGAATCTCAATTGCCAGGAATAAGGGAGGCTTTGGCATAAGTCAGAGACATGTTAGCTGGGTTAGTTCCAGAGGTTGTTTATTTGAGATTGAGAGATATGAA<br>TGAGAAGGAGATGCCCATCTAAGATTGGGTGCTGGTTTAGGTTTGGGAGATTGTGTATCCATTTAAAAAGGAAGGGGAATTTTAGAAA
	**RNAi resistant CEP90-GFP**	
**CEP90a-GFP RNAi resistant**		ATGAGTACGAAGAGAAAAGACATCATAAAACTTATGAAATCGATGAATATAGATCCAACCACATTTTCTGAGGAAGATATAAGATAATTTGAATAAGGCTAAGTGATGATGAGTGACAGTGAATCAGATAGTATGAGGGATTCGGTATCCATAGCTGATATTGAGAATTATCAATAGACTAAACATAACA<br>GAGGGGGGAACACTCATGATACAGTCAATATGAAAAATGTATTATTGTAATCGATCCATTTTTAGAATTATTCATCCGTTGTGGGCATGCAGGAAAAATAAATAGCTTTTCTAAATGCTGAGATCAAAAGGATCCAAGCAGCCAATGACGCTGATAGACAAAGAGTTTTTGCTAATCATCGAATTGAAACA<br>GATAAACTTTTAAGTGAAATCAAATAACTAAAGTCTAAGATGATCTATCAAGAGTCTCAATTACCAGGCATAAGAGAAGCTTTGGCTTAAGTTAGAGATATGCTTGCTGGATTGGTCCCAGAGGCTGTTTATCTGAGGTTGCGAGATATGAATGAGAAGGAAATGCCCATTCAAGATTGGGTATTGGTTTA<br>GGTTTGGGAAATTGTGTATCCATTCAAAAAGGAGGGGGAGTTTTAGAAAAAGGAAATATTGGCTTTAAGGGAAGAACTCAGATAGATAACAGAGAAGCATTAATTCTTATTGAATGATTTGGAACATGGCTAAAAGATGATGGTAGACAGAGAAGAGGATATTAGAAGACATAAATTAAATTATGATAAT<br>GCTAGAAAGGCTTTGGAGTTAGAATTAACAAAATCTTAAGAGGAATTAGCCCTGTTGAGAGAGAAGGGAGCAAGATTTGATGAATTAAGTAGGGATTATAAGAGATTGGAGTAAGAGAAGTTTTTATTGGAAGAGAAGGCTGGTTTCTATCAAAATAATTAAGCCGGAGACAAGGCAGGTGATTAATAC<br>AAGGCATAGGATGACGCCAAGAGGAAGGGAGACCTTTTGCAATAAGACAAGGAATATTTGACTAAGGAGAATATTTAACTTTTGGAGAAGGTTAAGAGATTGGAGGACAAATTAGATAGGACAGAGAAGGAGTATTTGGAAGCAAAAAACTAAGCTTAAGAATATCTATTTTAATTACTCAATTCTAAAA<br>CAGATTAGACTCAGGCTTATGAAAAAAGAATTTACTCAGAAATTGCAGATCTTAAAGAGAAGCATCATCATGAATTGGAAATTGCCAAATAGAATTTAGTTGATATTTATGAGACTCAAATCAAATTCTTGAAGGATGCAAAGGAGGAAATTTAACTTCGAAAAGATAATCTAGAAGGGCAAGTGAAGGA<br>AAAATCAACTTTATATGATCATTTGCTAATTGATTATAGAAACTTATAAAGAAAATTAGATGGGGACTTATCTGAATAGAGAATTCAATTAAGATTGAAGGTGGAAGAGTTGGATAGAGTATAGAATATCTATGAAGATA**CATTGGCTAATTTGAAAGCCACAAAACATGAAAACGAGATGTTGAGGG**<br>**AAAAAATCAATGTCTTAAAAGCCGAATATTATAAATGCTAAGTCGAGGCCAAGGAATAAATGTCAAGCATCAACGCTTAACTTTAAGTCGCCAAAGAATAATTGACTAACTATGAAGGAATCGAAAAGGAAATCGATGATGCTATTATGAAGTCAGCCGGAAACGAATACGATGCTATGA**<br>**ACCCTTTATTGGGATTCATGGGAACAGTCCCAACATCATCTAAAGGAGAATTCAATAAGCATTGAACCTCGCCTAAAGACTTTAAGCAAAGCAAAGAGAAAGCGAAGAGCTTTAAAGACAATTGAGAGACAAATCACAAGAAATCGAGAGACTTTAAGAGGAAACCAAATTACAAAGA**<br>**GATGTCTTGGATAAAACCCACTAACCTCACTCCTACCTCGTC**ATGAGTACGAAGAGAAAAGACATCATAAAACTTATGAAATCGATGAATATAGATCCAACCACATTTTCTGAGGAAGATATAAGATAATTTGAATAAGGCTAAGTGATGATGAGTGACAGTGAATCAGATAGTATGAGGGATTCGGT<br>ATCCATAGCTGATATTGAGAATTATCAATAGACTAAACATAACAGAGGGGGGAACACTCATGATACAGTCAATATGAAAAATGTATTATTGTAATCGATCCATTTTTAGAATTATTCATCCGTTGTGGGCATGCAGGAAAAATAAATAGCTTTTCTAAATGCTGAGATCAAAAGGATCCAAGCAGCCAATG<br>ACGCTGATAGACAAAGAGTTTTTGCTAATCATCGAATTGAAACAGATAAACTTTTAAGTGAAATCAAATAACTAAAGTCTAAGATGATCTATCAAGAGTCTCAATTACCAGGCATAAGAGAAGCTTTGGCTTAAGTTAGAGATATGCTTGCTGGATTGGTCCCAGAGGCTGTTTATCTGAGGTTGCGAGAT<br>ATGAATGAGAAGGAAATGCCCATTCAAGATTGGGTATTGGTTTAGGTTTGGGAAATTGTGTATCCATTCAAAAAGGAGGGGGAGTTTTAGAAAAAGGAAATATTGGCTTTAAGGGAAGAACTCAGATAGATAACAGAGAAGCATTAATTCTTATTGAATGATTTGGAACATGGCTAAAAGATGATGGTAG<br>ACAGAGAAGAGGATATTAGAAGACATAAATTAAATTATGATAATGCTAGAAAGGCTTTGGAGTTAGAATTAACAAAATCTTAAGAGGAATTAGCCCTGTTGAGAGAGAAGGGAGCAAGATTTGATGAATTAAGTAGGGATTATAAGAGATTGGAGTAAGAGAAGTTTTTATTGGAAGAGAAGGCTGGTT<br>TCTATCAAAATAATTAAGCCGGAGACAAGGCAGGTGATTAATACAAGGCATAGGATGACGCCAAGAGGAAGGGAGACCTTTTGCAATAAGACAAGGAATATTTGACTAAGGAGAATATTTAACTTTTGGAGAAGGTTAAGAGATTGGAGGACAAATTAGATAGGACAGAGAAGGAGTATTTGGAAGCAA<br>AAAACTAAGCTTAAGAATATCTATTTTAATTACTCAATTCTAAAACAGATTAGACTCAGGCTTATGAAAAAAGAATTTACTCAGAAATTGCAGATCTTAAAGAGAAGCATCATCATGAATTGGAAATTGCCAAATAGAATTTAGTTGATATTTATGAGACTCAAATCAAATTCTTGAAGGATGCAAAGGAG<br>GAAATTTAACTTCGAAAAGATAATCTAGAAGGGCAAGTGAAGGAAAAATCAACTTTATATGATCATTTGCTAATTGATTATAGAAACTTATAAAGAAAATTAGATGGGGACTTATCTGAATAGAGAATTCAATTAAGATTGAAGGTGGAAGAGTTGGATAGAGTATAGAATATCTATGAAGATACATTGG<br>CTAATTTGAAAGCCACAAAACATGAAAACGAGATGTTGAGGGAAAAAATCAATGTCTTAAAAGCCGAATATTATAAATGCTAAGTCGAGGCCAAGGAATAAATGTCAAGCATCAACGCTTAACTTTAAGTCGCCAAAGAATAATTGACTAACTATGAAGGAATCGAAAAGGAAATCGATGATGCTATTAT<br>GAAGTCAGCCGGAAACGAATACGATGCTATGAACCCTTTATTGGGATTCATGGGAACAGTCCCAACATCATCTAAAGGAGAATTCAATAAGCATTGAACCTCGCCTAAAGACTTTAAGCAAAGCAAAGAGAAAGCGAAGAGCTTTAAAGACAATTGAGAGACAAATCACAAGAAATCGAGAGACTTTAA<br>GAGGAAACCAAATTACAAAGAGATGTCTTGGATAAAACCCACTAACCTCACTCCTACCTCGTCGCCCATATTGAGGAAAAGGATAGAGAAATTTTGAAATATAAATCCCTTTTAAAGAAAATGGATTAGGATTATTAGATCCTAAAAGCAGAAAACGATGATGTAGCCAATGTAATTATTAGTGTATAAAT<br>TTAATAGAGATTCATGCGAGCTGAAGAGGATCTATAGAGATTGAGAATTAAAAGACAGAACCTACAAAACATTTAGAACATTTTGATTGGTATTGCCACTGGTAGTTAGAATGATATGAGATAAAATCTGAGAAGTGCCATAAATGAGATGTCGGAAGCCTTAAATACAAAGACATATATTGATCCTCAAA<br>GTGCGATCTAAAAAAAGGGTATGAATTTGCATTAATTGCAAGGTGGAATTCAAAGTAGTTAATAAAAATAGGAAGATGGTCCTTCTTGGTATTCAAAATTGAAACAAAAACAGAATAAAGGAGGAGGACTCGAGGTACGCATGTCTAAAGGAGAAGAATTATTCACTGGTGTTGTTCCAATTCTTGTTGA<br>ACTTGATGGTGATGTTAATGGACATAAATTTTCTGTTTCTGGTGAGGGTGAAGGTGATGCAACATATGGAAAATTAACCTTAAAATTTATTTGCACTACTGGAAAATTACCTGTTCCATGGCCAACACTTGTCACTACTTTAACTTATGGAGTTCAATGTTTTTCAAGATACCCTGATCATATGAAACAACAT<br>GACTTTTTCAAATCTGCCATGCCAGAAGGATATGTCCAAGAAAGAACTATATTCTTCAAAGATGATGGAAACTACAAGACAAGAGCTGAAGTCAAATTTGAAGGAGATACCCTTGTCAATAGAATTGAGTTAAAAGGAATTGATTTTAAAGAAGATGGAAACATTTTAGGACATAAATTGGAATACAACTA<br>TAACTCACATAATGTATACATCATGGCAGACAAACAAAAAAATGGAATCAAAGTCAACTTCAAAATTAGACACAACATTGAAGATGGATCAGTTCAATTAGCAGACCATTATCAACAAAATACTCCAATTGGAGATGGCCCTGTTTTATTACCAGACAACCATTACTTATCAACACAATCTGCCTTATCAAA<br>AGATCCAAATGAAAAGAGAGACCATATGGTTTTATTAGAGTTTGTAACTGCTGCTGGAATTACACATGGCATGGATGA
	**siRNA mammalian cells**	
**siRNA CEP90 #1**	siGENOME SMARTpool siRNA D-020125-01, PIBF1 Dharmacon	GAGAUUAGACAACCAAUUG
	siGENOME SMARTpool siRNA D-020125-02, PIBF1 Dharmacon	ACAGGUAUCUCAUUGAAUC
	siGENOME SMARTpool siRNA D-020125-03, PIBF1 Dharmacon	UCGUUAAGAUGCAUAGUAA
	siGENOME SMARTpool siRNA D-020125-04, PIBF1 Dharmacon	GAUCAGCUCUUAGACAGGU
**siRNA CEP90 #2**	based on siRNA CEP90#1 (Kodani *et al.* 2015) Dharmacon	TCTGCAAGAGGAAACAGCAAGAAAT
**siRNA FOPNL #1**	based on siRNA FOR20#2 (Sedjai et al. 2010) Dhamacon	GTATTATAAAGGCCCTTAA
**siRNA FOR20 #2**	siGENOME SMARTpool siRNA D-016243-01, FOPNL Dharmacon	GUACUAACAUUGAAGAUCU
	siGENOME SMARTpool siRNA D-016243-02, FOPNL Dharmacon	GCCAAUGGAUGACCACCUA
	siGENOME SMARTpool siRNA D-016243-03, FOPNL Dharmacon	GAUCCGAGCUGAAGUUUUC
	siGENOME SMARTpool siRNA D-016243-04, FOPNL Dhamacon	GCCAUUUCUUGCGUGGAA
**scrambled control**	siGENOME Non-Targeting siRNA Pool #2 Dharmacon	UAAGGCUAUGAAGAGAUAC
		AUGUAUUGGCCUGUAUUAG
		AUGAACGUGAAUUGCUCAA
		UGGUUUACAUGUCGACUAA
