# Supplementary figures and images for: The evolutionary conserved proteins CEP90, FOPNL, and OFD1 recruit centriolar distal appendage proteins to initiate their assembly
Source: PLoS Biol. 2022 Sep 7;20(9):e3001782. doi: 10.1371/journal.pbio.3001782 (PMC9484695; doi:10.1371/journal.pbio.3001782)

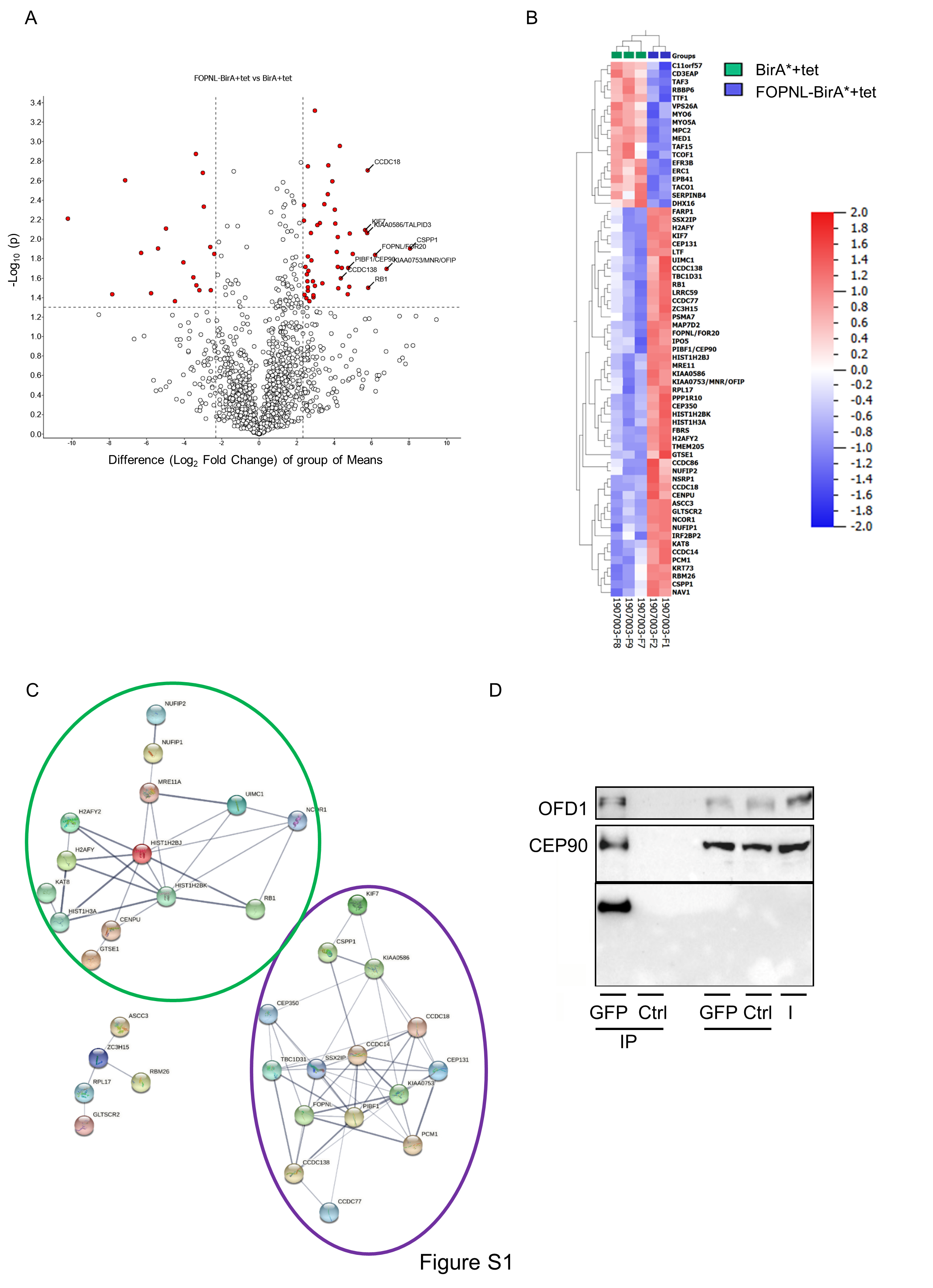

Supplement: S1 Fig — (A). VolcanoPlot of proteins identified in FOPNL-myc-BirA* + tet versus myc-BirA*+ tet, made with Qlucore. The X-axis shows the LOG2 of the Fold Change and the Y-axis shows the -LOG10 of the p-value derived from a Student bilateral t test assuming equal variance between groups. Differential genes satisfying both p-value <0.05 and Fold Change > 5 were colored in red. (B) Heatmap of proteins identified in myc-BirA*-FOPNL + tet versus myc-BirA*+ tet, made with Qlucore. Proteins were filtered based on a p-value < 0.05 and Fold Change > 5, intensities were log2 transformed and were displayed as colours ranging from red to blue. Both rows and columns were clustered hierarchically. Source data can be found in S7 Data. (C) Protein–protein interaction network from differentially abundant proteins identified in FOPNL-BirA+tet versus BirA*+tet. Proteins were filtered based on a p-value <0.05 and Fold Change >5. The network was created using STRING DB representing a full STRING network at medium confidence (0.400) with disconnected nodes hidden. Lines of different thicknesses between nodes symbolize the Edge confidence from medium to the highest. The cluster encircled in purple indicates centriolar and centriolar satellite proteins. The one in green corresponds to nuclear proteins. (D) Co-immunoprecipitation experiment made in HeLa Kyoto GFP-FOPNL. Cell extract was immunoprecipitated with mouse control or anti-GFP immunoglobulins. The input, the unbound fraction (FT) as well as the bound fraction (IP) were revealed with either rabbit polyclonal anti-GFP, CEP90, and OFD1 antibodies. Both CEP90 and OFD1 are co-immunoprecipitated by GFP antibodies, indicating a complex between FOPNL, OFD1 and CEP90. tet, tetracyclin. (TIF) [file pbio.3001782.s003.TIF]

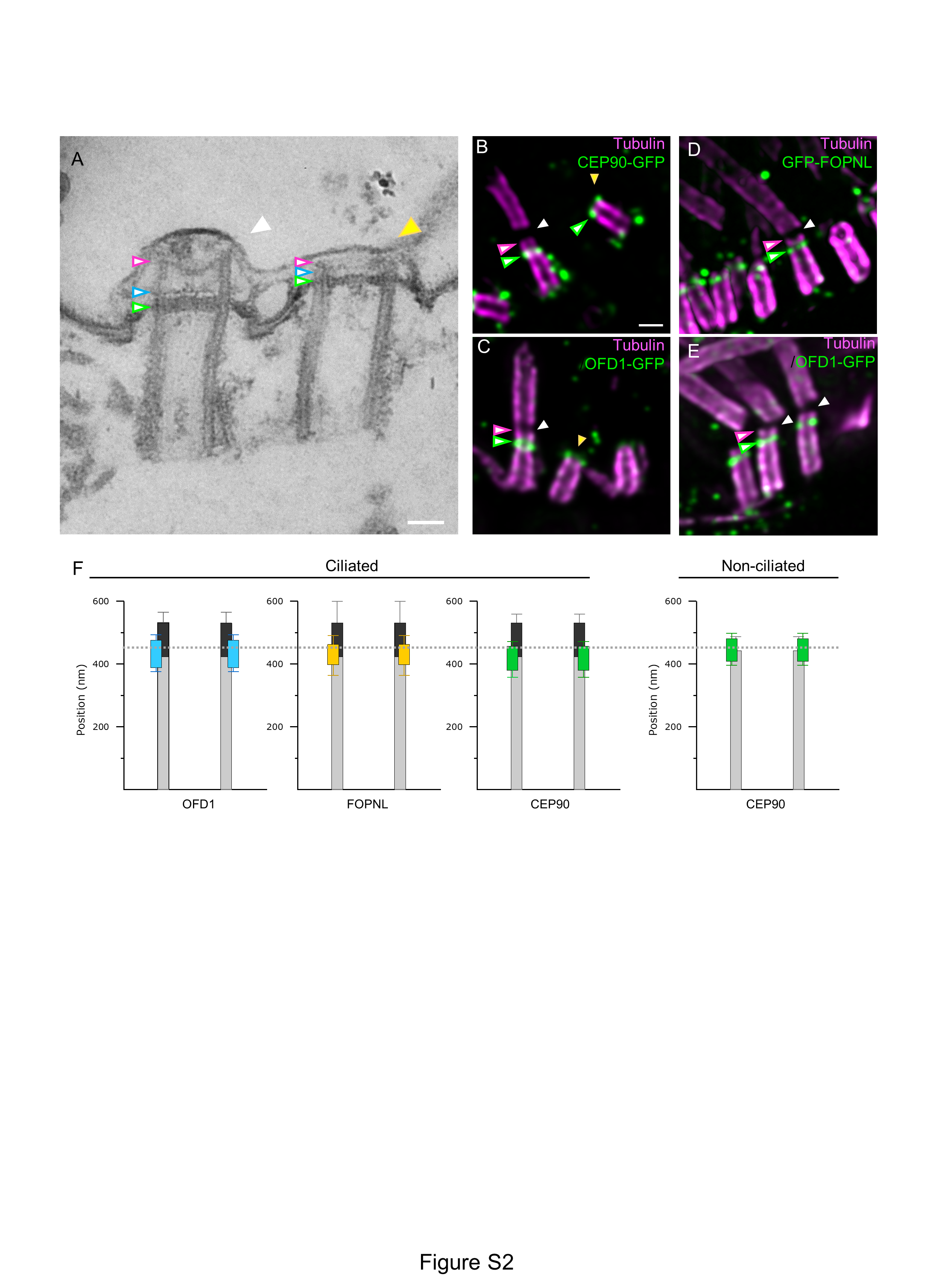

Supplement: S2 Fig — (A) EM on Paramecium cortex fragment showing a 2-BB unit. During cortex purification, cilia shed above the TZ (white arrowhead). The unciliated BB (yellow arrowhead) remain unmodified. Scale bar = 100 nm. (B-E) U-ExM performed on paramecia transformants expressing CEP90-GFP (B), OFD1-GFP (C, E), and GFP-FOPNL (D) show BB and cilia stained with tubulin antibody (magenta). Note that a space indicated by a white arrowhead shows the cilium breakage between the distal end of the TZ and the cilium. (B, C) CEP90-GFP and OFD1-GFP signals are localized at the distal extremity of nonciliated BB (yellow arrowhead), while GFP signal is slightly underneath the distal end of ciliated BB (white arrowhead). Scale bar = 250 nm. (F) Schematic representation of OFD1 (blue square), FOPNL (yellow square), and CEP90 (green square) localization on ciliated and unciliated BB. Scale bar = 200 nm. Source data can be found in S8 Data. BB, basal body; TZ, transition zone; U-ExM, ultrastructure expansion microscopy. (TIF) [file pbio.3001782.s004.TIF]

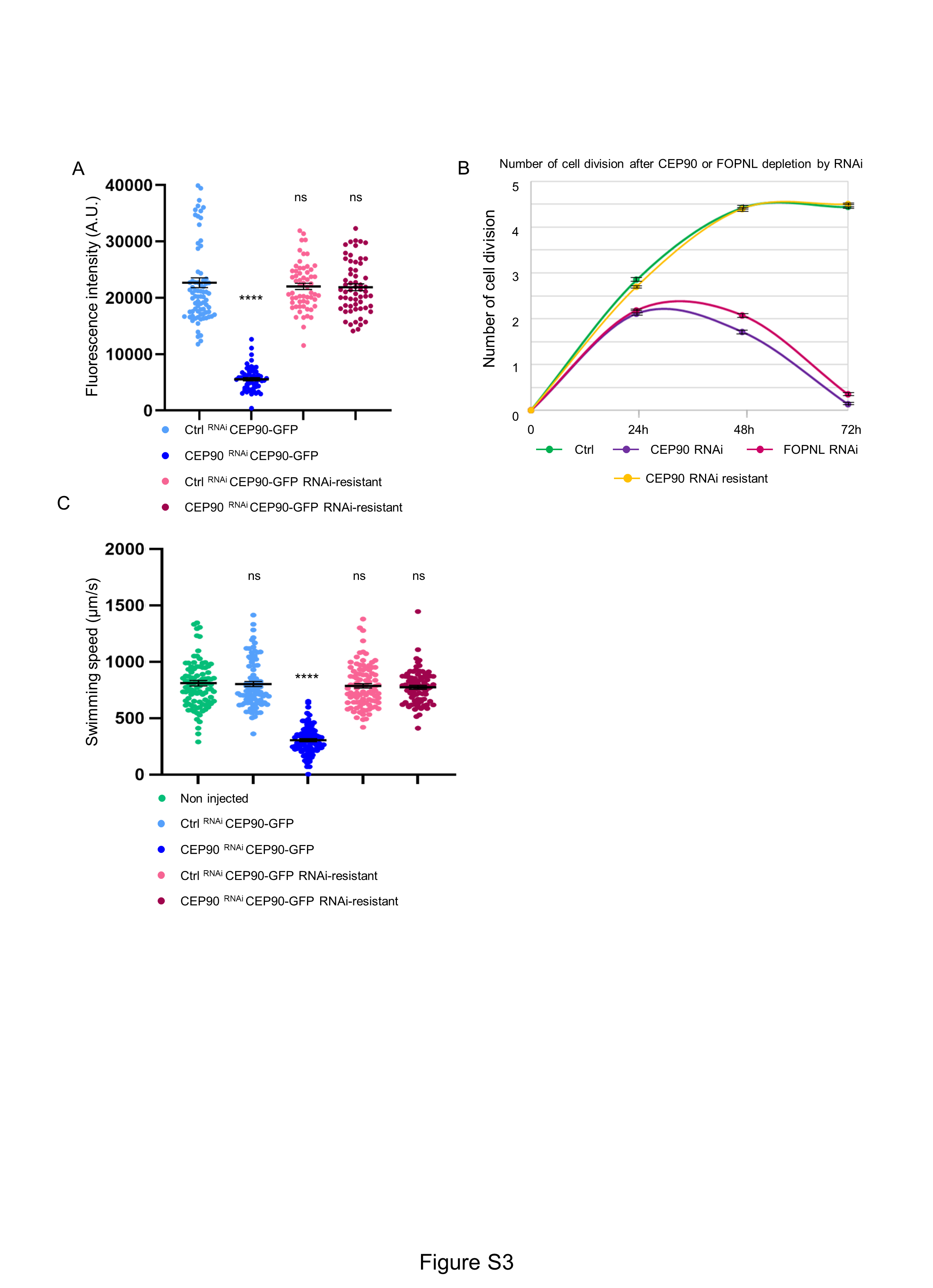

Supplement: S3 Fig — (A-C): Paramecium were transformed with a construction expressing either WT CEP90-GFP or an RNAi-resistant CEP90-GFP. (A) Quantification of BB GFP signal fluorescence on newly formed BB (anterior ones) after 2 or 3 divisions upon CEP90 depletion. average ± SEM is represented, ****p < 0.001 (one-way ANOVA followed by Tukey’s post hoc test), n ≥ 60 BB from 2 independent experiments. Source data can be found in S9 Data. (B) Number of cell divisions in control, CEP90, and FOPNL depleted cells as well as in cells rescued with RNAi-resistant CEP90. Note that both CEP90 and FOPNL depleted cells died after 4 or 5 divisions. Source data can be found in S9 Data. (C) Quantification of the swimming speeds of noninjected paramecia (NI), paramecia expressing WT CEP90-GFP, or RNAi-resistant CEP90-GFP after 48 h under control condition or CEP90 RNAi condition. Each dot shows the average velocity of 1 cell, average ± SEM is represented, ****p < 0.001 (one-way ANOVA followed by Tukey’s post hoc test, n ≥ 60 cells per condition performed from 2 independent experiments). Source data can be found in S9 Data. BB, basal body; RNAi, RNA interference; WT, wild-type. (TIF) [file pbio.3001782.s005.TIF]

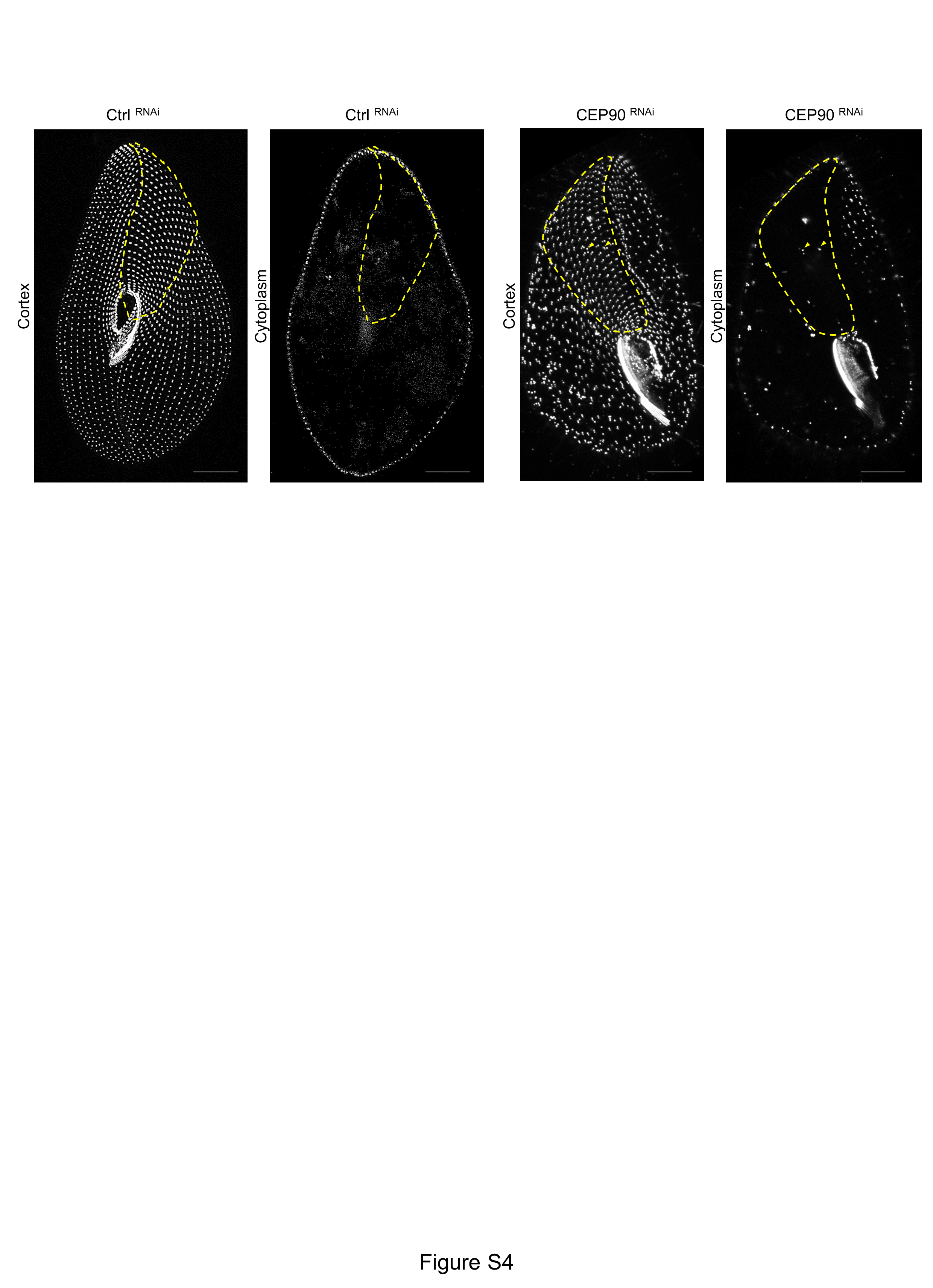

Supplement: S4 Fig — (A) Early stage of Paramecium division stained with poly-E antibodies (grey). Cells were observed after control (CtrlRNAi) and CEP90 depletion (CEP90RNAi) after the first division. The posterior part of the cell shows defective BB organization in CEP90-depleted cell, suggesting that the RNAi has been effective. The invariant field encircled in yellow on the figure shows the usual 2 BB pattern at the cortical level, suggesting that BB duplication has occurred normally. Defective BB duplication will lead to only 1 BB in this field. Sometimes additional BB are found but appear below the cortical surface (yellow arrowheads). Scale bars = 20 μm. BB, basal body; RNAi, RNA interference. (TIF) [file pbio.3001782.s006.TIF]

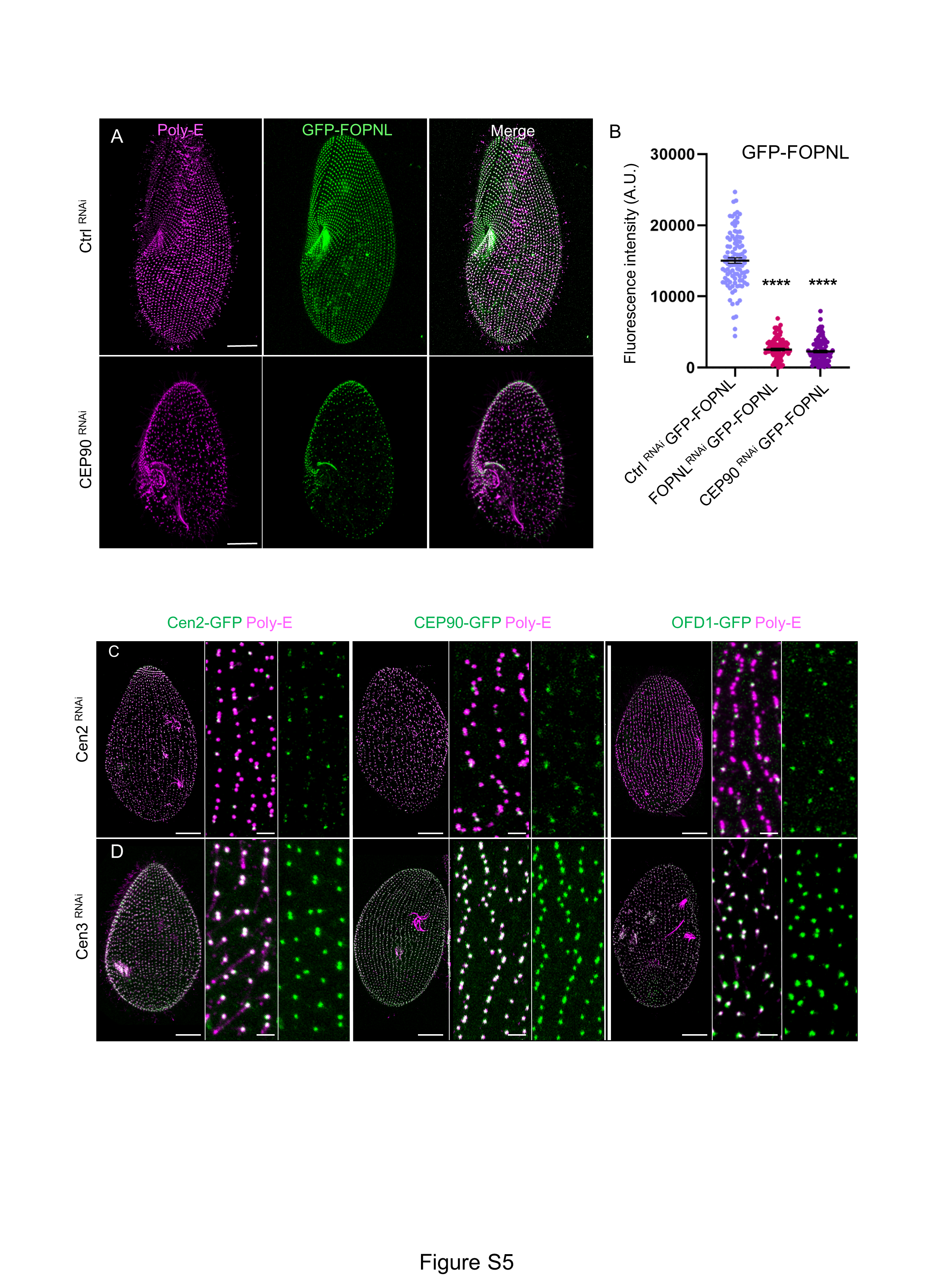

Supplement: S5 Fig — (A) Transformants expressing a GFP-tagged FOPNL were observed 2–3 divisions upon control (Ctrl) or CEP90 depletion. GFP-FOPNL fluorescence (green) was severely reduced on newly formed BB along with the expected BB pattern disorganization. Scale bar = 20 μm. (B) Dot plots showing the average percentage of GFP-FOPNL BB fluorescence after 2–3 divisions in either FOPNL or CEP90 RNAi (n ≥ 100 BB per condition performed in 2 independent replicates). Error bars show SEM. Statistical significance was assessed by a one-way ANOVA followed by Tukey’s post hoc test p < 0.0001 ****. Source data can be found in S10 Data. (C, D) Paramecium transformants expressing Cen2-GFP, CEP90 GFP, or OFD1-GFP (green) have been stained for BB (poly-E antibodies, magenta) after 2–3 divisions upon Cen2 (C) or Cen3 (D) depletion. After Cen2 and Cen3 depletion, BB pattern disorganization is observed. (C) The GFP fluorescence is not recruited to newly formed BB after Cen2 RNAi. (D) By contrast, Cen3 depletion did not affect neither CEP90-GFP, OFD1-GFP, and GFP-FOPNL recruitment to BB. Scale bars = 20 μm and 2 μm (magnification). AU, arbitrary units; BB, basal body; RNAi, RNA interference. (TIF) [file pbio.3001782.s007.TIF]

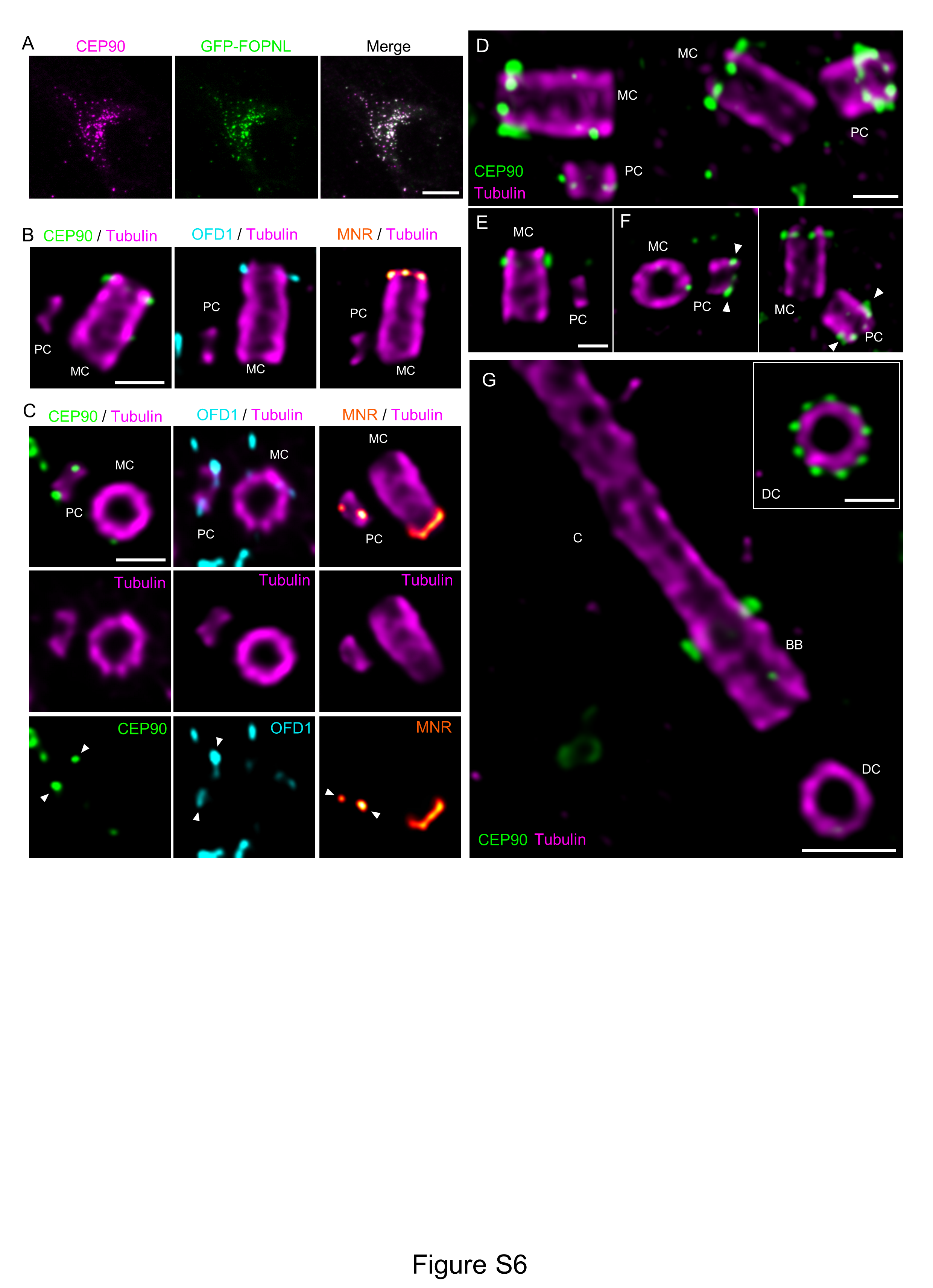

Supplement: S6 Fig — (A) HeLa-Kyoto cell lines expressing GFP-FOPNL (green) were stained for CEP90 (magenta). Confocal images show the colocalization of both proteins at centrosome and centriolar satellites. Scale bar = 5 μm. (B, C) Confocal images of duplicating U2OS cell, expanded and stained for tubulin (magenta) and CEP90 (green), OFD1 (cyan), or MNR (orange/red) showing the recruitment of the 3 proteins at early stages of procentriole assembly, slightly after the arrival of the tubulin (PC: procentriole, MC: mature centriole). Scale bars: 200 nm. (D-F) Confocal images of duplicating RPE1 cells, expanded and stained for tubulin (magenta) and CEP90 (green) showing the presence of CEP90 slightly underneath the distal end of both the mother (MC) and procentrioles (PC). Note that CEP90 is recruited at early stages of procentriole assembly just after the start of the tubulin (arrowheads) Scale bar = 200 nm. (G) Confocal images of ciliated RPE1 cell, expanded and stained for tubulin (magenta) and CEP90 (green) showing the presence of CEP90 at the distal part of the BB templating the primary cilium (C). Please note that the daughter centriole (DC) is also positive for CEP90 as shown in the upright insert where the distal part of the DC is shown. Scale bar = 200 nm. BB, basal body; DC, daughter centriole; MC, mature centriole; MNR, Moonraker; PC, procentriole. (TIF) [file pbio.3001782.s008.TIF]

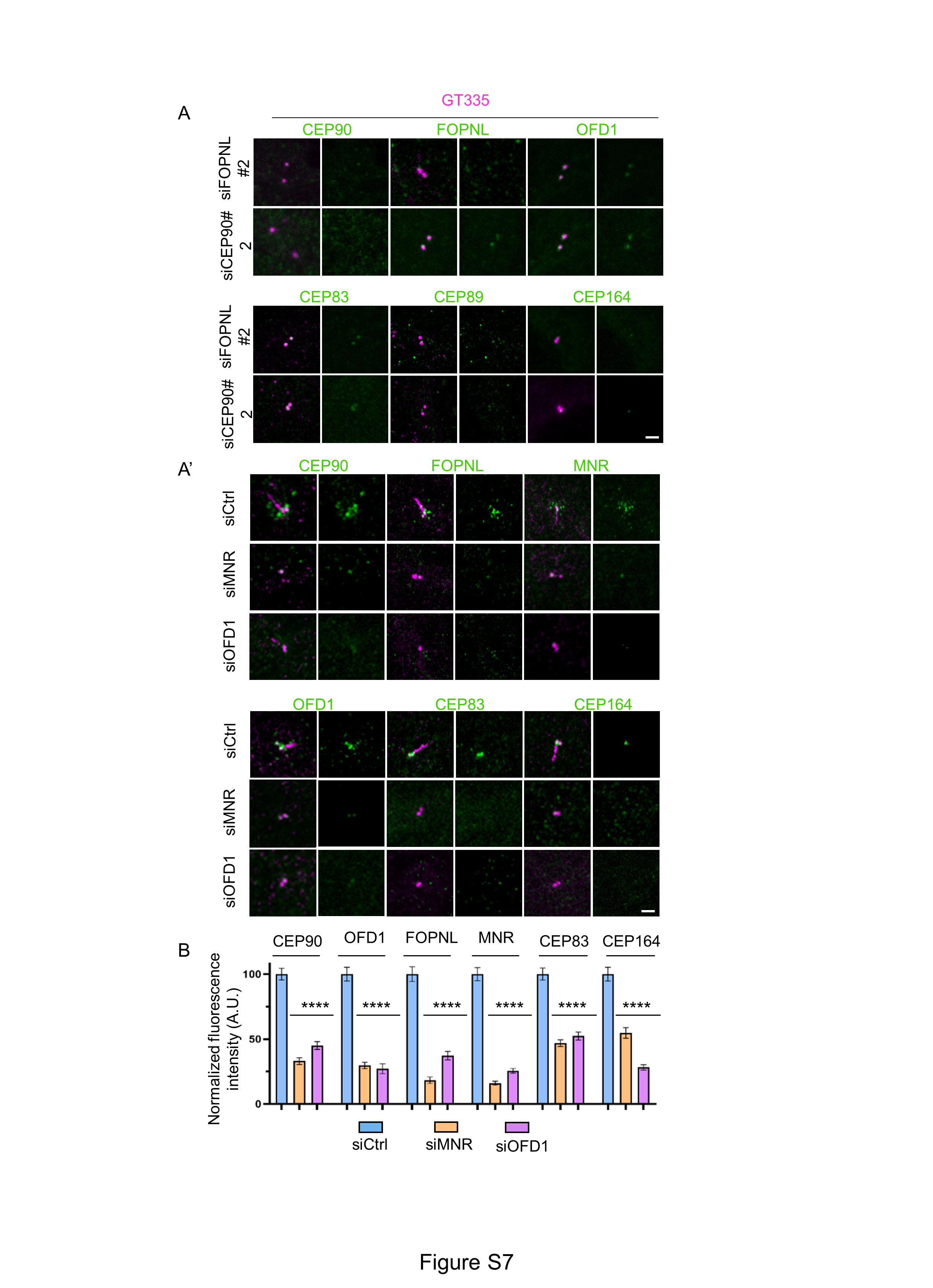

Supplement: S7 Fig — (A) Representative images of distal appendage proteins labelling after depletion of CEP90 (siRNA#2) or FOPNL (siRNA#2), stained with CEP90, FOPNL, OFD1, CEP83, CEP89, CEP164 antibodies (green). The centrosome is stained with GT335 (magenta). Control siRNA and CEP90 (siRNA#1) and FOPNL (siRNA#1) are found in Fig 6A. (B) Quantification of the fluorescence intensity of antibodies: CEP90, OFD1, MNR, FOPNL, CEP83, and CEP164 staining at the centrosome. All data are presented as average ± SEM. ****p < 0.001 (one-way ANOVA followed by Tukey’s post hoc test), n ≥ 100 centrosomes in 3 independent experiments. Source data can be found in S11 Data. AU, arbitrary units. (TIF) [file pbio.3001782.s009.TIF]

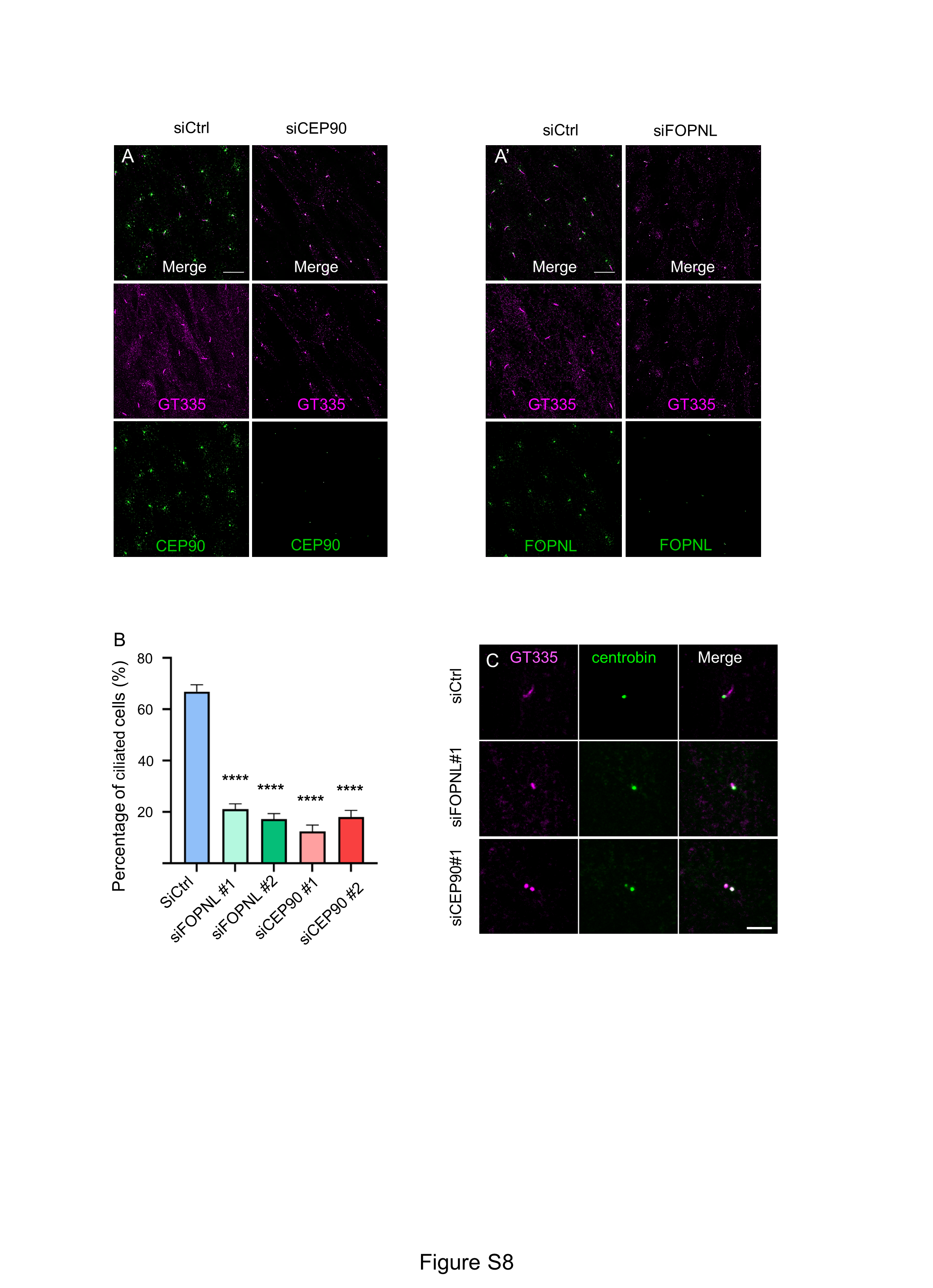

Supplement: S8 Fig — (A, A’) Serum-starved RPE1 during 24 h in control (siCtrl) condition and upon CEP90 (A) or FOPNL (A’) depletion were stained for GT335 (magenta) and CEP90 (A, green) or FOPNL (A’, green). Scale bars: 15 μm. (B) Percentage of ciliated cells were quantified in each condition. Average ± SEM is represented, ****p < 0.001 (one-way ANOVA followed by Tukey’s post hoc test, n ≥ 350 cells per condition performed in 2 independent replicates). Source data can be found in S12 Data. (C) The daughter centriolar protein Centrobin was localized in control (siCtrl), CEP90, and FOPNL-depleted RPE1 cells. No significant differences are observed between control-depleted cells and CEP90 or FOPNL-depleted cells. Scale bar = 5 μm. (TIF) [file pbio.3001782.s010.TIF]

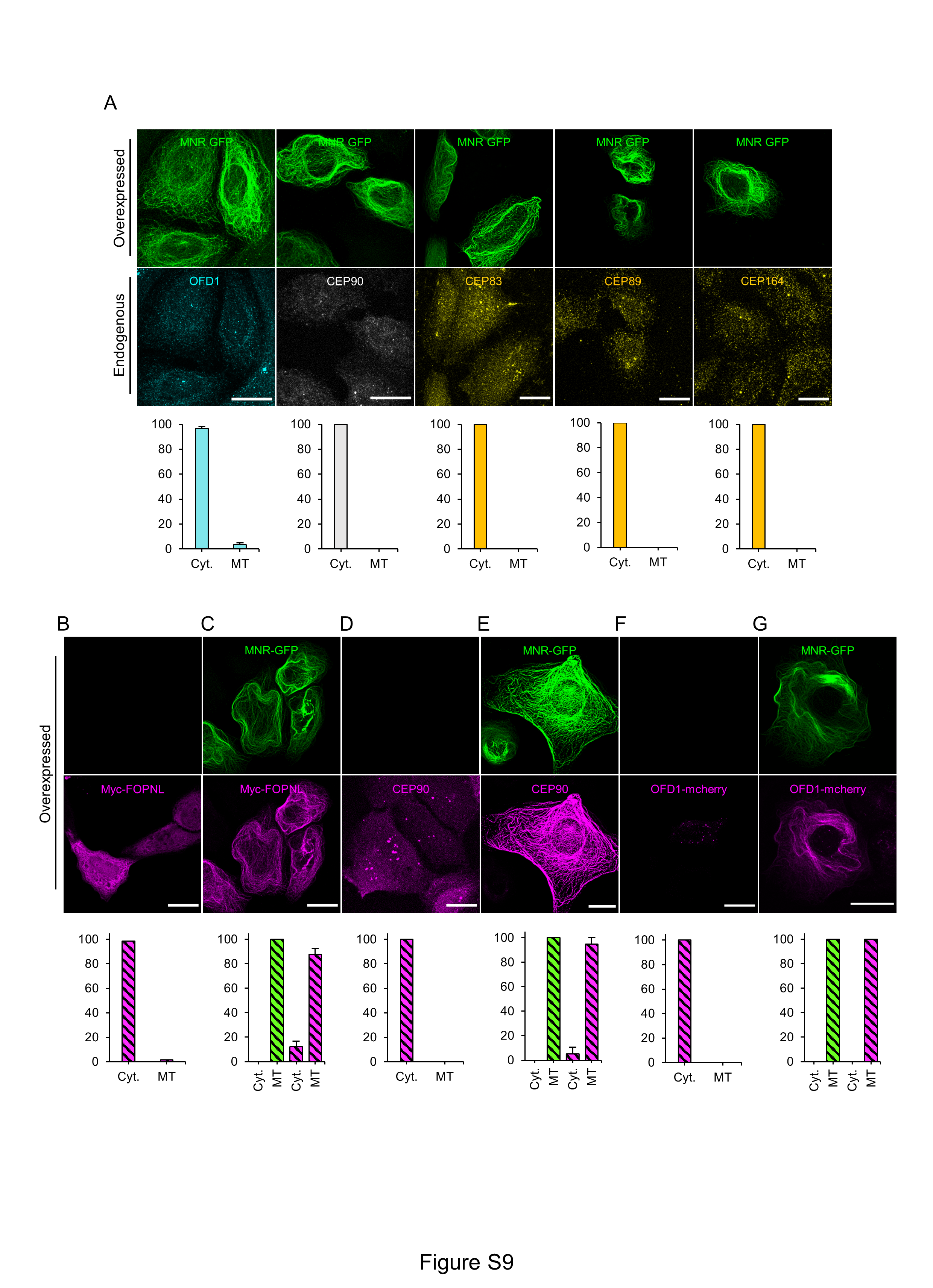

Supplement: S9 Fig — (A) U2OS cells overexpressing MNR-GFP stained for endogenous OFD1, CEP90, CEP83, CEP89, and CEP164 as well as the percentage of cells displaying endogenous proteins to either Cyt or MT. Scale bars: 20 μm. Source data can be found in S13 Data. (B, D, F) Overexpression of myc-FOPNL, CEP90, and OFD1 mcherry and their localization to the cytoplasm as well as the percentage of cells displaying the localization of proteins to either Cyt or MTs. (C, E, G) Co-overexpression of myc-FOPNL, CEP90, and OFD1 mcherry with MNR-GFP. Note that the cytoplasmic localization of myc-FOPNL, CEP90, and OFD1 mcherry shift with MNR on the microtubules. Percentage of cells displaying the localization of proteins to either Cyt or MTs. Scale bars = 20μm. Source data can be found in S13 Data. Cyt, cytoplasm; MNR, Moonraker; MT, microtubule. (TIF) [file pbio.3001782.s011.TIF]
